# cAMP signaling inhibits radiation-induced ATM phosphorylation leading to the augmentation of apoptosis in human lung cancer cells

**DOI:** 10.1186/1476-4598-13-36

**Published:** 2014-02-24

**Authors:** Eun-Ah Cho, Eui-Jun Kim, Sahng-June Kwak, Yong-Sung Juhnn

**Affiliations:** 1 Department of Biochemistry and Molecular Biology, Cancer Research Institute, Seoul National University College of Medicine, Seoul 110-799, Korea; 2 Department of Biochemistry, College of Medicine, Dankook University, Chonan, 330–714, Korea

**Keywords:** cAMP signaling, ATM, Protein phosphatase 2A, Apoptosis, NF-κB, Lung cancer

## Abstract

**Background:**

The ataxia–telangiectasia mutated (ATM) protein kinase plays a central role in coordinating the cellular response to radiation-induced DNA damage. cAMP signaling regulates various cellular responses including metabolism and gene expression. This study aimed to investigate the mechanism through which cAMP signaling regulates ATM activation and cellular responses to ionizing radiation in lung cancer cells.

**Methods:**

Lung cancer cells were transfected with constitutively active stimulatory G protein (GαsQL), and irradiated with γ-rays. The phosphorylation of ATM and protein phosphatase 2A was analyzed by western blotting, and apoptosis was assessed by western blotting, flow cytometry, and TUNNEL staining. The promoter activity of NF-κB was determined by dual luciferase reporter assay. BALB/c mice were treated with forskolin to assess the effect in the lung tissue.

**Results:**

Transient expression of GαsQL significantly inhibited radiation-induced ATM phosphorylation in H1299 human lung cancer cells. Treatment with okadaic acid or knock down of PP2A B56δ subunit abolished the inhibitory effect of Gαs on radiation-induced ATM phosphorylation. Expression of GαsQL increased phosphorylation of the B56δ and PP2A activity, and inhibition of PKA blocked Gαs-induced PP2A activation. GαsQL enhanced radiation-induced cleavage of caspase-3 and PARP and increased the number of early apoptotic cells. The radiation-induced apoptosis was increased by inhibition of NF-κB using PDTC or inhibition of ATM using KU55933 or siRNA against ATM. Pretreatment of BALB/c mice with forskolin stimulated phosphorylation of PP2A B56δ, inhibited the activation of ATM and NF-κB, and augmented radiation-induced apoptosis in the lung tissue. GαsQL expression decreased the nuclear levels of the p50 and p65 subunits and NF-κB-dependent activity after γ-ray irradiation in H1299 cells. Pretreatment with prostaglandin E2 or isoproterenol increased B56δ phosphorylation, decreased radiation-induced ATM phosphorylation and increased apoptosis.

**Conclusions:**

cAMP signaling inhibits radiation-induced ATM activation by PKA-dependent activation of PP2A, and this signaling mechanism augments radiation-induced apoptosis by reducing ATM-dependent activation of NF-κB in lung cancer cells.

## Background

Radiotherapy is one of the major treatment modalities for benign and malignant diseases throughout the body. Approximately 50% of all cancer patients are treated with radiotherapy, and there is a wide inter-patient variability in tumor responses. Strategies to improve radiotherapy seek to increase the effects of radiation on the tumor or decrease the effects on normal tissues. An improved understanding of the molecular response of cells and tissues to ionizing radiation has contributed to improvements in radiotherapy [1]. Ionizing radiation can induce single-strand breaks (SSBs) and double-strand breaks (DSBs) in the DNA double helix backbone that trigger DNA damage responses. The DNA damage response machinery delays cell cycle progression and activates cell cycle checkpoints to provide more time for lesion repair and prevent the transfer of damaged DNA to progeny. When repair fails, the damaged cells are commonly eliminated from the proliferative pool through cellular senescence or several types of cell death, including apoptosis [2].

Together with ataxia–telangiectasia and RAD3-related (ATR) and DNA-dependent protein kinase catalytic subunit (DNA-PKcs), the ataxia–telangiectasia mutated (ATM) protein kinase plays a central role in coordinating the cellular response to DNA damage [3-5]. Deficiency in the ATM kinase causes ataxia-telangiectasia, a rare autosomal recessive disorder characterized by hypersensitivity to radiation and predisposition to cancer. ATM belongs to the phosphatidylinositol 3 kinase-like kinase (PIKK) family of Ser/Thr-protein kinases, which contains ATR, DNA-PKcs and mTOR (mammalian target of rapamycin) [6]. Following DNA damage, an intermolecular autophosphorylation occurs on Ser-1981 of ATM that disrupts the inactive homodimer and enables the kinase domain to phosphorylate several target substrates and trigger downstream signaling pathways [5]. Many ATM substrates regulate gene expression, cell-cycle checkpoints, DNA repair and apoptosis [7]. Thus, ATM is a potential target molecule for the development of novel radiosensitizers [8,9].

Cyclic adenosine 3', 5'-monophosphate (cAMP) is a second messenger that is produced from ATP by adenylate cyclases and degraded into 5’-AMP by cyclic nucleotide phosphodiesterases. Adenylate cyclase is activated by stimulatory heterotrimeric GTP-binding proteins (G proteins), which are activated by G protein coupled receptor (GPCR)-agonist complexes [10]. cAMP binds to and activates the cAMP-dependent protein kinase (PKA), the cAMP-activated guanine exchange factors (Epacs), which are the guanine nucleotide exchange factors (GEFs) for monomeric G protein Raps [11], and the cyclic nucleotide-gated channels functioning in transduction of sensory signals (CNGs). The cAMP signaling system regulates numerous cellular responses including gene expression, growth, differentiation, proliferation, and apoptosis.

We have reported that the cAMP signaling system modulates cancer cell apoptosis by regulating the expression of Bcl-2 family proteins [12,13] and the inhibitor of apoptosis protein (IAP) [14] in response to various DNA damaging agents, including ionizing radiation. Recently, the cAMP signaling system was found to inhibit the repair of γ-ray-induced DNA damage by promoting degradation of the XRCC1 protein in human lung cancer cells [15]. The cAMP signaling system was also reported to inhibit DNA-damage induced apoptosis of leukemia cells by promoting acetylation and turnover of p53 [16,17]. Thus, we hypothesized that the cAMP signaling system might be involved in the regulation of ATM activation, the key event triggering signaling pathways in response to DNA damage. This study aimed to investigate the mechanism through which the cAMP signaling system regulates ATM activation and cellular responses following γ-ray irradiation. We found that Gαs inhibits ATM activation via the Gαs-cAMP-PKA-PP2A pathway and augments radiation-induced apoptosis following γ-ray irradiation in non-small cell lung cancer cells.

## Results

### *G*αs inhibited radiation-induced ATM activation in lung cancer cells

**Figure 1:**
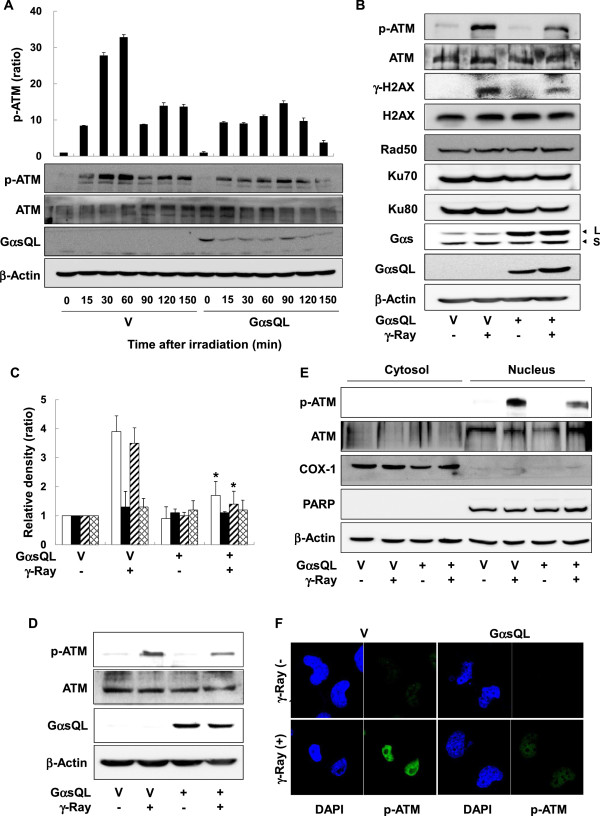
**Gαs inhibited ATM activation induced by γ−ray irradiation in lung cancer cells. (A)** Effect of Gαs on the phosphorylation of ATM following γ-ray irradiation in H1299 lung cancer cells. **(B)** Effect of Gαs on the proteins involved in DNA damage responses following γ-ray irradiation in H1299 cells. L represents the long forms of Gαs, and S represents the short forms of Gαs. **(C)** Densitometric analysis of the phosphorylation of ATM and H2AX. The histograms represent the means and standard errors of at least three independent experiments (empty bar: p-ATM, filled bar: ATM, diagonal bar: γ-H2AX, and hatched bar: H2AX), and an asterisk (*) indicates a statistically significant difference from the vector-transfected control cells (p < 0.05, Mann–Whitney U test). **(D)** Effect of Gαs on the phosphorylation of ATM following γ-ray irradiation in A549 lung cancer cells. **(E)** Subcellular fractionation analysis of ATM phosphorylation. **(F)** Confocal microscopic analysis of ATM phosphorylation. Lung cancer cells (H1299 and A549 cells) were transfected with EE-tagged GαsQL or a pcDNA3 vector (V), incubated for 24 h, and irradiated with γ-rays (5 Gy). After incubation for 30 min or for the indicated times, the expression and phosphorylation of the proteins involved in DNA damage responses were analyzed by western blotting. Each lane represents cells that were separately transfected, and β-actin was used as a loading control. Thirty minutes after irradiation, the cells were lysed and fractionated for western blotting. COX-1 and PARP were used as markers for cytosolic and nuclear fractions, respectively. One hour after irradiation, phosphorylated ATM was assessed by staining with p-ATM-FITC (green) and DAPI (blue), and the samples were then analyzed by confocal microscopy.

To investigate the effects of cAMP signaling on radiation-induced DNA damage responses, an EE-tagged constitutively active mutant long form of the α subunit of stimulatory heterotrimeric GTP binding protein (GαsQL) was transiently expressed in H1299 human lung cancer cells. Irradiation of H1299 cells with γ-rays induced a biphasic phosphorylation of ATM: ATM phosphorylation started at 15 min after irradiation and reached peak levels at 30 min, followed by a second peak at 120 min. Expression of GαsQL decreased the peak level of ATM phosphorylation at 30 min and displayed the initial peak at 90 min after irradiation (Figure 1A & Additional file 1: Figure S1). GαsQL expression significantly inhibited the radiation-induced phosphorylation of ATM and H2AX 30 min after γ-ray irradiation in H1299 cells, without changing their protein levels; the expression of Rad50, Ku70, and Ku80 also remained unchanged (Figure 1B). The densitometric analyses of the blots confirmed the decrease in ATM and H2AX phosphorylation by GαsQL (Figure 1C). GαsQL expression also inhibited the radiation-induced phosphorylation of ATM in A594 lung cancer cells (Figure 1D). Both western blot analysis of the subcellular fractions (Figure 1E) and confocal microscopic analysis (Figure 1F) showed that Gαs inhibited radiation-induced ATM activation in the nucleus within 1 h after γ-ray exposure. Furthermore, to confirm the inhibition of ATM activity by Gαs, the effect of Gαs on ATM downstream target molecules: p53 and CHK2 was analyzed. GαsQL expression decreased radiation-induced phosphorylation of p53 in A549 cells, and CHK2 in H1299 and A549 cells (Additional file 1: Figure S2). In addition, treatment with N6-benzoyl cAMP, a PKA selective cAMP analogue, also inhibited radiation-induced ATM phosphorylation (Additional file 1: Figure S3). These results show that Gαs inhibits radiation-induced ATM activation at the early phase of the DNA damage response in lung cancer cells.

### *G*αs activated PP2A in a PKA-dependent manner, causing the inhibition of radiation-induced phosphorylation of ATM in H1299 lung cancer cells

**Figure 2:**
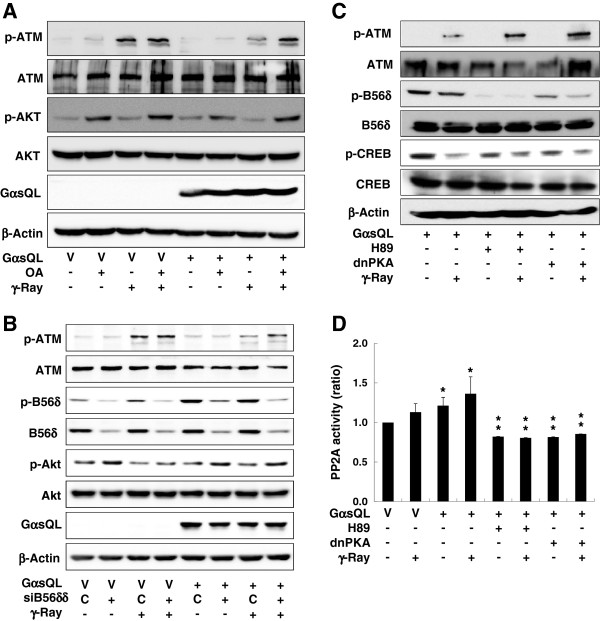
**Gαs activated PP2A in a PKA dependent manner, which decreased the radiation-induced phosphorylation of ATM in H1299 lung cancer cells. (A)** Effect of okadaic acid (OA) on the radiation-induced ATM phosphorylation. **(B)** Effect of Gαs on PP2A B56δ phosphorylation. **(C)** Effect of PKA inhibition on the phosphorylation of PP2A B56δ and ATM. **(D)** Effect of Gαs on PP2A activity. H1299 cells were transfected with GαsQL, vector (V), or dominant negative PKA (dnPKA) and incubated for 24 h. siRNA against B56δ (siB56δ) or control siRNA was transfected and the cells were incubated for 48 h before the treatment. The cells were pretreated with 500 nM okadaic acid, 10 μM H89, or DMSO as a vehicle for 30 min, and then the cells were irradiated with γ-rays (5 Gy). After incubation for 30 min, the cells were harvested and analyzed by western blotting and for PP2A activity. Phosphorylated AKT (p-AKT) was analyzed as a positive control for PP2A activity. Asterisks (*) on the histograms indicate a statistically significant difference from the vector-transfected control cells; the double asterisks (**) represent a statistically significant difference from the GαsQL-transfected control cells (p < 0.05, Mann–Whitney U test, D).

To investigate the mechanism through which Gαs inhibited radiation-induced ATM phosphorylation, the effect of a PP2A inhibitor, okadaic acid, on ATM phosphorylation was analyzed. It was because the phosphorylation level of proteins including ATM is regulated by both the protein kinases and phosphatases, and because ATM is not as a known PKA substrate but known to be dephosphorylated by PP2A which is activated by PKA. Treatment with okadaic acid abolished the inhibitory effect of Gαs on radiation-induced ATM phosphorylation and recovered the phosphorylation to the control level in the GαsQL-transfected cells (Figure 2A & Additional file 1: Figure S5). Then, to examine whether Gαs could activate PP2A, the phosphorylation of the PP2A B56δ subunit at Ser-566 was analyzed in GαsQL-transfected cells. Expression of GαsQL strongly increased the basal phosphorylation level of the B56δ subunit, and the increased B56δ subunit phosphorylation was maintained after irradiation without an observable change in the protein level (Figure 2B & Additional file 1: Figure S6). Furthermore, knockdown of PP2A B56δ subunit with siRNA abolished the inhibitory effect of Gαs on radiation-induced ATM phosphorylation (Fig 2B & Additional file 1: Figure S6). Next, to determine if phosphorylation of the PP2A B56δ subunit by Gαs was catalyzed by PKA, the effect of PKA inhibition was assessed. Inhibition of PKA with the inhibitor H89 or a dominant negative PKA decreased the phosphorylation of PP2A B56δ before and after γ-ray irradiation and resulted in a concomitant increase in ATM phosphorylation (Figure 2C & Additional file 1: Figure S7). The effective inhibition of PKA by H89 or a dominant negative PKA was evidenced by a decrease in phosphorylated CREB, which is a well-known PKA target protein. Then, the effect of Gαs signaling on PP2A enzyme activity was analyzed. Expression of GαsQL increased PP2A activity before and after γ-ray irradiation compared with the respective control, and the PP2A-activating effect of Gαs was completely blocked by H89 or the dominant negative PKA (Figure 2D). These results indicate that Gαs activates PP2A by phosphorylating the B56δ subunit in a PKA-dependent manner, which decreases radiation-induced phosphorylation of ATM in H1299 lung cancer cells.

### *G*αs augmented radiation-induced apoptosis by inhibiting ATM activation in lung cancer cells and mouse lung tissue

**Figure 3:**
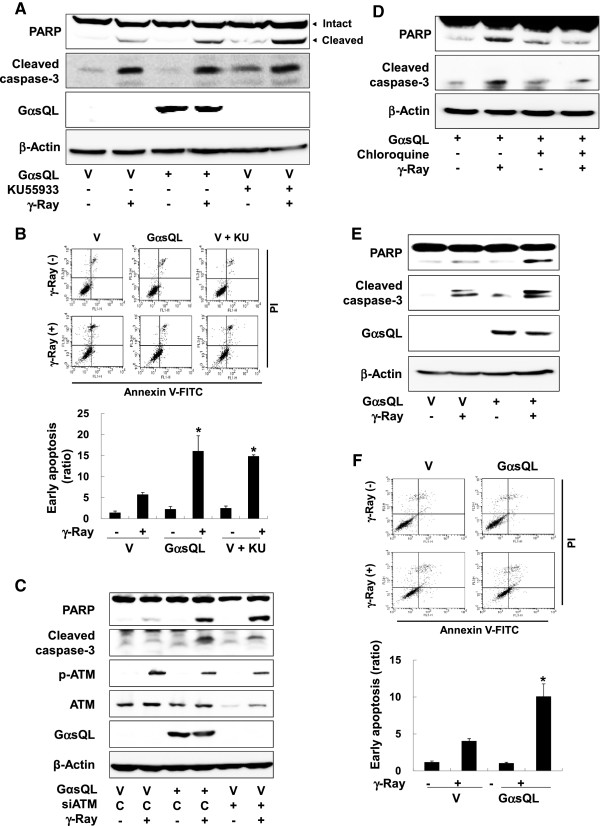
**Gαs augmented radiation-induced apoptosis by inhibiting ATM activation in lung cancer cells. (A)** Effects of Gαs on radiation-induced cleavage of caspase-3 and PARP in H1299 cells. **(B)** Effects of Gαs on radiation-induced early apoptosis in H1299 cells. **(C)** Effects of ATM knockdown on radiation-induced cleavage of caspase-3 and PARP in H1299 cells. **(D)** Effect of chloroquine on radiation-induced apoptosis in H1299 cells. **(E)** Effect of GαsQL on radiation-induced cleavage of caspase 3 and PARP in A549 cells. **(F)** Effects of Gαs on radiation-induced early apoptosis in A549 cells. The cells were irradiated with γ-rays (5 Gy) and incubated for 30 min for analysis of ATM phosphorylation. For analysis of apoptosis, the cells were pretreated with 10 μM KU55933 or DMSO for 30 min, irradiated with γ-rays (10 Gy) and incubated for 24 h. To determine the effects of chloroquine, the cells were pretreated with 20 μg/ml chloroquine for 4 h before irradiation; the culture medium was replaced with fresh medium at 1 h after irradiation. The cells were incubated for an additional 23 h, and then harvested and analyzed by western blotting or flow cytometry after staining with annexin V and propidium iodide (PI). The histograms present the ratio of annexin V-positive but propidium iodide-negative cells, and asterisks (*) on the histograms indicate a statistically significant difference from the vector-transfected control cells (p < 0.05, Mann–Whitney U test).

To investigate the physiological effects of the inhibition of radiation-induced ATM activation by Gαs, we examined the effect on radiation-induced apoptosis. In H1299 cells, expression of GαsQL enhanced radiation-induced cleavage of caspase 3 and PARP (Figure 3A). GαsQL expression also increased the number of cells stained with annexin V but not with propidium iodide following irradiation (Figure 3B & Additional file 1: Figure S8), and decreased survival of irradiated cells in clonogenic assay (Additional file 1: Figure S9). Treatment with an ATM inhibitor, KU55933, also enhanced the radiation-induced cleavage of caspase 3 and PARP and increased the proportion of annexin V-stained cells (Figure 3A & B). Knockdown of ATM with siRNA also enhanced the radiation-induced cleavage of caspase 3 and PARP (Figure 3C). In contrast, activation of ATM by pretreatment with chloroquine decreased the radiation-induced cleavage of caspase 3 and PARP (Figure 3D). In addition, A549 human lung cancer cells were used to confirm that the observed effects of Gαs also occurred in other lung cancer cells. Expression of GαsQL in A549 cells also enhanced the radiation-induced cleavage of caspase 3 and PARP and increased the number of annexin V stained cells (Figure 3E & F). These results indicate that Gαs augments the radiation-induced apoptosis by inhibiting ATM activation in human lung cancer cells.

**Figure 4:**
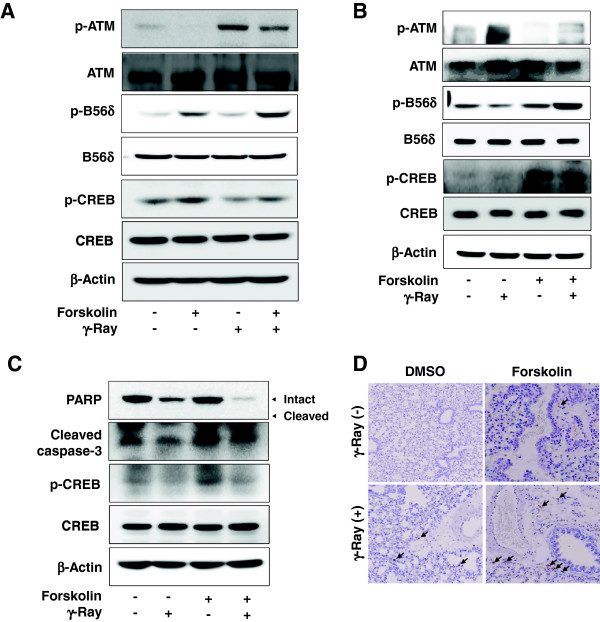
**Forskolin augmented radiation-induced apoptosis by inhibiting ATM activation in the mouse lung.(A)** Effects of forskolin on the phosphorylation of PP2A B56δ and ATM in B16F10 mouse melanoma cells. Mouse melanoma cells were pretreated with 40 μM forskolin for 30 min and irradiated with γ-rays (5 Gy). After incubation for 30 min, the phosphorylation of PP2A B56δ and ATM were analyzed by western blotting. **(B)** Effects of forskolin on the phosphorylation of PP2A B56δ and ATM in the mouse lung. **(C)** Effect of forskolin on apoptosis in the mouse lung analyzed by western blotting. **(D)** Effect of forskolin on apoptosis in the mouse lung analyzed by the TUNEL assay. Four-week-old male BALB/c mice (20 g, n = 6) were injected intraperitoneally with forskolin (20 μg/g) or DMSO, and after 6 h, the mice were exposed to whole body γ-ray irradiation (10 Gy). After incubation for 30 min, the animals were sacrificed, and the lung tissues were excised and homogenized for western blot analysis of ATM and PP2A. For apoptosis analysis, the animals were sacrificed at 24 h after irradiation, and the lung tissues were excised. A portion of the lung tissues were homogenized and analyzed for cleavage of caspase-3 and PARP by western blotting. The other portion of the tissue was immediately fixed with formaldehyde and examined by the TUNEL assay. The arrows indicate the stained cells undergoing apoptosis.

Next, BALB/c mice were used to verify the effect of Gαs activation in vivo. Prior to the animal experiment, the effect of forskolin, an adenylate cyclase activator similar to Gαs, was analyzed in B16-F10 mouse melanoma cells. Treatment with forskolin increased the radiation-induced phosphorylation of the PP2A B56δ subunit and decreased the radiation-induced phosphorylation of ATM in the melanoma cells (Figure 4A). Pretreatment of BALB/c mice with forskolin stimulated phosphorylation of PP2A B56δ and inhibited the phosphorylation of ATM in lung tissue following γ-ray irradiation (Figure 4B). Furthermore, forskolin treatment of BALB/c mice increased radiation-induced apoptosis in the lung tissue as evidenced by an increased cleavage of caspase-3 and PARP (Figure 4C) and an increase in TUNEL-stained cells following γ-ray irradiation (Figure 4D). These results suggest that cAMP signaling augments radiation-induced apoptosis by inhibiting ATM activation via PP2A in mouse lung, as well as in human lung cancer cells and murine melanoma cells.

### *G*αs augmented radiation-induced apoptosis by reducing ATM-dependent activation of NF-κB

**Figure 5:**
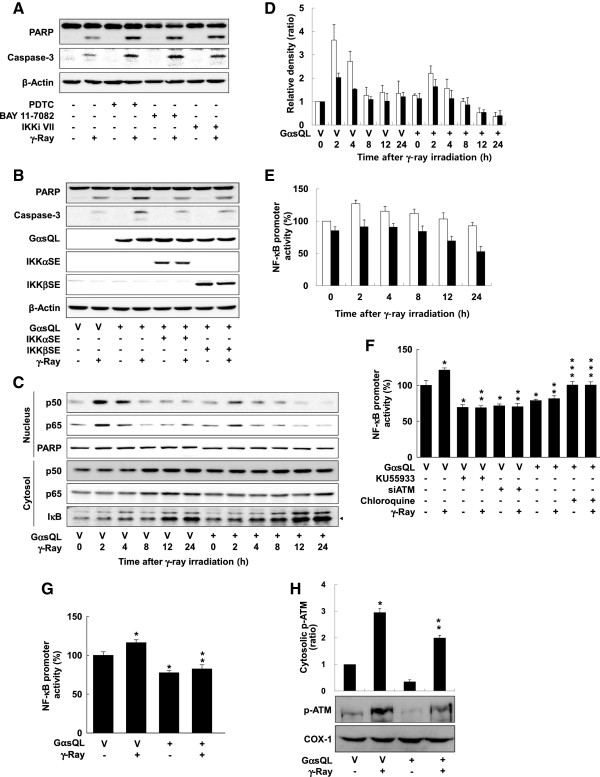
***Reduced ATM-dependent activation of NF-κB augmented radiation-induced apoptosis by G*α*s.*(A)** Effect of NF-κB inhibitors on radiation-induced cleavage of caspase 3 and PARP in H1299 cells. **(B)** Effect of constitutively active IKKα (IKKαSE) and IKKβ (IKKβSE) on on radiation;-induced cleavage of caspase 3 and PARP in H1299 cells. **(C, D)** Effect of Gαs on the activation of NF-κB in H1299 cells. The graph was made from the western blot band densities (empty bar: nuclear p50, filled bar: nuclear p65). **(E)** Effect of Gαs on NF-κB-dependent luciferase activity in H1299 cells. The empty bar represents vector control and the filled bar GαsQL transfected cells. **(F)** Effect of ATM activity on NF-κB-dependent luciferase activity in H1299 cells. **(G)** Effect of Gαs on NF-κB-dependent luciferase activity in A549 cells **(H)** Effect of Gαs on cytosolic pATM. Asterisks (*) on the histograms indicate a statistically significant difference from the vector-transfected control cells; the double asterisks (**) represent a statistically significant difference from the irradiated control cells; the triple asterisks (***) represent a statistically significant difference from the GαsQL-transfected control cells (p < 0.05, Mann–Whitney U test). H1299 cells were pretreated with KU55933 (10 μM), or PDTC (5 μM), BAY 11–7082 (10 μM), or IKK inhibitor VII (IKKi VII, 500 μM) for 30 min or chloroquine (20 μg/ml) for 4 h with/without transfection of GαsQL or vector. Then, the cells were irradiated with γ-rays (10 Gy) and incubated for 24 h. NF-κB-dependent luciferase activity was assessed using the Dual-Luciferase Reporter Assay System. The cells were also lysed and fractionated into nucleus and cytosol fraction before western blot analysis. PARP was the markers for nucleus fraction.

To study the mechanism through which reduced ATM activation augments radiation-induced apoptosis, we examined the role of NF-κB, which is known to be activated by ATM to prevent apoptosis [18]. Inhibition of NF-κB by treatment with NF-κB inhibitors such as PDTC, BAY 11–7082, and IKK inhibitor VII increased the radiation-induced cleavage of caspase-3 and PARP in H1299 cells (Figure 5A). Furthermore, activation of NF-κB by expression of constitutively active IKKα and IKKβ reduced the cleavage of caspase-3 and PARP augmented by GαsQL (Figure 5B), indicating inhibition of NF-κB activity augments radiation-induced apoptosis. Next, the effect of radiation and Gαs on NF-κB activation was examined. Radiation increased nuclear translocation of NF-kB p50 and p65 with a peak at 2 h after irradiation, and the expression of GαsQL reduced the radiation-induced translocation of p50 and p65 (Figure 5C & D). Then, the effect on NF-κB-dependent promoter activity was analyzed. Radiation slightly increased NF-κB-dependent promoter activity, and the expression of GαsQL reduced the promoter activity until 24 h after irradiation (Figure 5E). Next, the role of ATM in NF-κB activation was assessed. Inhibition of ATM activation by treatment with an ATM inhibitor, KU55933, or by knockdown with siRNA reduced the NF-κB-dependent promoter activity before and 2 h after irradiation (Figure 5F). Activation of ATM by pretreatment with chloroquine abolished the reducing effect of GαsQL on NF-κB-dependent promoter activity (Figure 5F). The expression of GαsQL also reduced the NF-κB activity before and after irradiation in A549 lung cancer cells (Figure 5G). These results suggest that Gαs augments radiation-induced apoptosis by reducing ATM-dependent activation of NF-κB in lung cancer cells.

To probe the mechanism how ATM activate NF-kB after irradiation, we determined the effect of Gαs on the level of phosphorylated ATM in the cytosol, where IκBα is located and degraded following phosphorylation. Although most of the phosphorylated ATM is localized in the nucleus, a small amount of phosphorylated ATM in the cytosol could be visualized after γ-ray irradiation by exposing blots to the gel documentation system for a longer period of time. γ-Ray irradiation increased the amount of phosphorylated ATM in the cytosol, and GαsQL expression decreased the amount of phosphorylated ATM in the cytosol following irradiation (Figure 5H). This result indicates that Gαs reduced the translocation of phosphorylated ATM into the cytosol, which might decrease phosphorylation and degradation IκBα protein and reduce activation of NF-κB in H1299 lung cancer cells.

### Prostaglandin E2 and isoproterenol affected ATM activation and apoptosis similarly to Gαs

**Figure 6:**
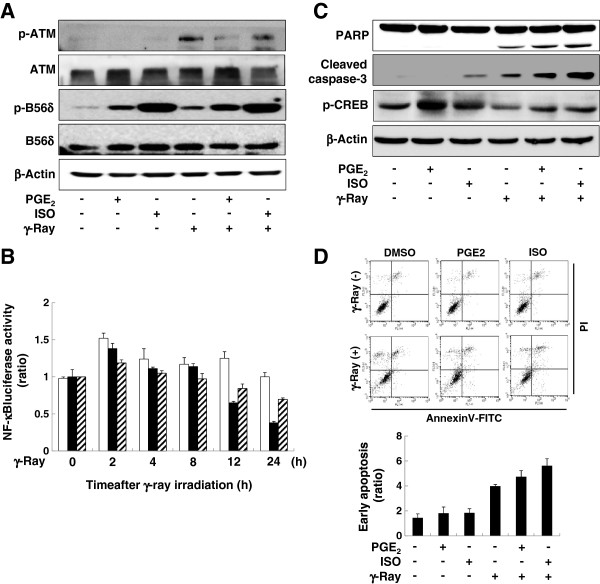
**Agonists for Gαs-coupled receptors inhibited ATM phosphorylation and augmented apoptosis following γ-ray irradiation in H1299 cells. (A)** Effects of prostaglandin E2 (PGE2) and isoproterenol (ISO) on PP2A B56δ and ATM phosphorylation. **(B)** Time-course of NF-κB luciferase activity after prostaglandin E2 or isoproterenol treatment. **(C)** Effects of prostaglandin E2 and isoproterenol on the cleavage of caspase 3 and PARP. **(D)** Effects of prostaglandin E2 and isoproterenol on early apoptosis. H1299 cells were treated with 1 μM isoproterenol or 10 μM PGE2 for 30 min before irradiation with γ-rays (5 Gy for or 10 Gy for others). Cells were then incubated for 30 min for analysis of phosphorylated ATM, B56δ, and CREB or 24 h for apoptosis analysis. For NF-κB luciferase activity assays, cells transfected with the reporter genes were treated with 1 μM isoproterenol or 10 μM PGE2 at30 min before irradiation (10 Gy), and the luciferase activity was measured at the indicated times (empty bar: control cells, filled bar: PGE2-treated cells, diagonal bar: isoproterenol-treated cells).

**Figure 7:**
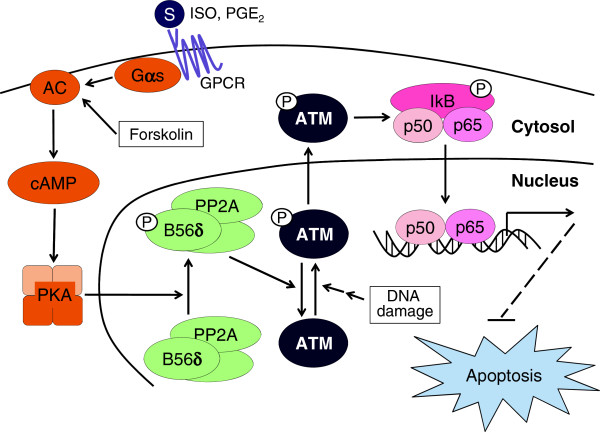
A suggested model for cAMP signaling to inhibit radiation-induced ATM activation, which results in augmentation of apoptosis in lung cancer.

To confirm the effects observed upon GαsQL expression, we analyzed the effects of prostaglandin E2 and isoproterenol, two agonists for Gαs-coupled receptors. Pretreatment with prostaglandin E2 and isoproterenol increased the phosphorylation of PP2A B56δ and decreased ATM phosphorylation following γ-ray irradiation (Figure 6A). Pretreatment with prostaglandin E2 decreased NF-κB luciferase activity 12 h after irradiation and the activity was not recovered until 24 h after irradiation. Isoproterenol treatment showed a similar inhibitory effect on radiation-induced NF-κB-dependent promoter activity (Figure 6B). The inhibitory effect of prostaglandin E2 and isoproterenol on ATM phosphorylation was abolished by treatment with a PKA inhibitor, H-89 (Additional file 1: Figure S10). Prostaglandin E2 or isoproterenol treatments also enhanced the cleavage of caspase-3 and PARP (Figure 6C & Additional file 1: Figure S11) and increased the proportion of early apoptotic H1299 cells (Figure 6D). Moreover, treatment with prostaglandin E2 significantly decreased survival of the irradiated cells (Additional file 1: Figure S12). These results indicate that agonists for Gαs-coupled receptors can activate PP2A and inhibit ATM and NF-κB similar to Gαs and, thus, augment apoptosis following γ-ray irradiation in H1299 cells (Figure 7).

## Discussion

This study aimed to investigate the mechanism through which the cAMP signaling system might regulate the activation of ATM and apoptosis following γ-ray irradiation. We found that cAMP signaling inhibits radiation-induced activation of ATM by PKA-dependent activation of PP2A, and the cAMP signaling system augments radiation-induced apoptosis partially by reducing the ATM-dependent activation of NF-κB in human lung cancer cells and mouse lung.

Our finding that the cAMP signaling system inhibits radiation-induced activation of ATM by PKA-dependent activation of PP2A is supported by several results. First, radiation-induced phosphorylation of ATM was inhibited by expression of constitutively active Gαs and by treatment with Gαs-coupled receptor agonists or an adenylate cyclase activator, forskolin. Second, treatment with a PP2A inhibitor or knock down of PP2A B56δ subunit abolished the ATM-inhibitory effect of Gαs. Third, expression of the active Gαs increased the phosphorylation of the PP2A B56δ subunit and enhanced PP2A activity. In addition, inhibition of PKA abolished the PP2A activation induced by Gαs, thereby restoring ATM phosphorylation. Moreover, inhibition of radiation-induced ATM phosphorylation by the cAMP signaling system was observed in human lung cancer cells, murine melanoma cells, and murine lung tissue, suggesting that the inhibition occurs in many tissues.

ATM is primarily recruited to double-strand DNA breaks and activated through interactions with the MRE11–RAD50–NBS1 (MRN) complex [19]. ATM protein undergoes autophosphorylation at Ser-1981 and forms monomers from an inactive dimer following double-strand DNA breaks; ATM autophosphorylation is considered a hallmark of ATM activation [20]. Recently, ATM was found to be activated independently from DNA damage through redox-dependent mechanisms and to participate in diverse signaling pathways involved in metabolic regulation and cancer [5]. However, no previous reports show that the cAMP signaling system regulates radiation-induced activation of ATM. Caffeine is known to inhibit ATM activation and has been studied as a potential radioenhancer [8]. Caffeine is also known to inhibit cAMP phosphodiesterase, which may increase the cAMP level [21], suggesting the involvement of the cAMP signaling system in ATM activation. However, caffeine was reported to inhibit the enzymatic activity of ATM immunoprecipitates in vitro, which was interpreted as direct inhibition of ATM by caffeine [22], independent of the cAMP signaling system. Thus, to the best of our knowledge, this paper presents the first evidence that the cAMP signaling system can regulate radiation-induced ATM activation.

PP2A-mediated inhibition of ATM activation in a PKA-dependent pathway is supported by the previous report that PKA phosphorylates Ser-566 of the PP2A B56δ subunit to stimulate PP2A activity [23]. PP2A forms complexes with ATM and dephosphorylates the autophosphorylated Ser-1981 in undamaged cells to suppress the intrinsic ATM activity [24].

This study shows that the cAMP signaling system augments radiation-induced apoptosis by inhibiting ATM activation. This finding is based on the result that radiation-induced apoptosis was augmented by the activation of the cAMP signaling system and by inhibition of ATM with a specific inhibitor, KU55933, and siRNA against ATM in cancer cells and mouse lung. In addition, the cAMP signaling system inhibits radiation-induced activation of ATM. This finding is supported by the fact that ATM is a master regulator of cellular responses to DNA damage caused by ionizing radiation and activates downstream signaling pathways to regulate various DNA damage responses including cell cycle, DNA repair, and apoptosis [25,26]. This finding suggests that cAMP signaling can modulate radiation-induced apoptosis by regulating radiation-induced ATM activation. This finding also implies that drugs targeting the cAMP signaling pathway could be possibly used to modulate radiation-induced apoptosis, thereby increasing the radiosensitivity of cancer cells or protecting normal cells from radiation. The cAMP signaling system can stimulate or inhibit apoptosis depending on cell types [27] through diverse molecular mechanisms involving Bcl-2 family proteins, p53, and histone deacetylase [16,28,29]. Thus, this study presents a novel mechanism for the cAMP signaling system to regulate cancer cell apoptosis. It is also plausible that the cAMP signaling system modulates other cellular responses to DNA damage mediated by ATM, such as DNA damage repair and cell cycle arrest.

The cAMP signaling system was found to augment radiation-induced apoptosis partly by inhibiting ATM-mediated NF-κB activation in this study. This finding is substantiated by the result that activation of the cAMP signaling system or inhibition of ATM resulted in a reduction of radiation-induced NF-κB activation and augmentation of apoptosis. In addition, inhibition of NF-κB activation by treatment with several NF-κB specific inhibitors augmented radiation-induced apoptosis, but activation of NF-κB signaling by expression of constitutively active IKKs abolished apoptosis-augmenting effect of cAMP signaling system. ATM can stimulate NF-κB activation, which induces the expression of anti-apoptotic proteins to protect cells from apoptosis. Thus, inhibition of ATM may compel the cells to undergo apoptosis as observed in this study [30,31]. However, ATM can play contrasting roles in DNA damage-induced apoptosis, and ATM induces apoptosis by phosphorylating downstream target substrates such as p53, TRF1 [32] and NBS1 [33]. Therefore, ATM seems to show different apoptotic effects depending on the cell type, DNA damage-inducing agent, the severity of DNA damage, and the presence of functional p53 [34].

NF-κB is activated in response to various immune and inflammatory stimuli, and it is also activated by ionizing radiation to protect damaged cells from apoptotic cell death [35,36]. The signal transduction mechanisms that link DNA damage to NF-κB activation are relatively unknown, but signaling pathways involving ATM and NF-κB essential modulator (NEMO) are reported to cooperate to directly link DNA damage in the nucleus to NF-κB activation in the cytosol [37]. ATM is involved in the sequential post-translational modification of NEMO, and ATM translocates in a calcium-dependent manner to the cytosol and membrane [38]. Cytosolic ATM activates TGFβ-activated kinase (TAK1), which phosphorylates IKKβ to trigger ubiquitin-proteasome dependent degradation of IκB and NF-κB activation [18]. In agreement with these findings, the cAMP signaling system was observed to reduce the cytosolic translocation of phosphorylated ATM accompanied with increased IκB level following γ-ray irradiation in this study, which may have resulted from inhibition of radiation-induced ATM phosphorylation and could cause reduced NF-κB activation and augmented apoptosis.

In this study, the role of the cAMP signaling system in ATM, PP2A and NF-κB activation, as well as in apoptosis, following γ-ray irradiation was assessed by activating the signaling system using various mechanisms: expression of constitutively active Gαs, treatment with Gαs-coupled receptor agonists such as isoproterenol for β-adrenergic receptors and prostaglandin E2 for prostanoid receptors, or treatment with the adenylate cyclase activator forskolin. Furthermore, similar effects were observed in A549 and p53-null H1299 human lung cancer cells, murine melanoma cells, and murine lung tissue, suggesting comparable effects of the cAMP signaling system in various cells and tissues. These results reinforce the inhibitory role of the cAMP pathway in radiation-induced activation of ATM by PKA-dependent activation of PP2A. These findings also suggest the augmentation of radiation-induced apoptosis potentially through a reduction of ATM-dependent NF-κB activation.

## Conclusion

The cAMP signaling system inhibits radiation-induced activation of ATM by PKA-dependent activation of PP2A, thereby augmenting radiation-induced apoptosis in part by reducing ATM-dependent activation of NF-κB in lung cancer cells and mouse lung tissue (Figure 7). These findings provide a novel mechanism through which the cAMP signaling system regulates radiation-induced ATM activation and apoptosis, and these findings suggest that the cAMP signaling system can be used to modulate DNA damage responses to enhance the therapeutic efficiency of radiation treatment for non-small cell lung cancers.

## Methods

### Cell culture and reagents

Human non-small cell lung cancer cell lines H1299 and A549 (Korea Cell Line Bank, Seoul, Korea) and B16-F10 mouse melanoma cells (ATCC, Manassas, VA, USA) were cultured in Dulbecco’s modified Eagle’s medium (DMEM) containing 10% fetal bovine serum (JBI, Korea) and 100 units/ml penicillin/streptomycin. The cells were incubated in a 5% CO_2_ incubator at 37°C. H89, isoproterenol, dimethyl sulfoxide (DMSO), and 4,6-diamidino-2-phenylindole dihydrochloride (DAPI) were purchased from Sigma (St. Louis, MO, USA). Forskolin, pyrrolidine dithiocarbamate (PDTC), IKK inhibitor VII, BAY 11–7082 and isobutylmethylxanthine (IBMX) were purchased from Calbiochem (La Jolla, CA, USA). The FITC Annexin V apoptosis detection kit was purchased from BD Biosciences (San Diego, CA, USA). Prostaglandin E2 (PGE2) and okadaic acid were purchased from Cayman Chemical (Ann Arbor, MI, USA). KU-55933 was purchased from Selleck Chemicals (Houston, TX, USA). Bovine serum albumin (BSA) and goat anti-rabbit IgG-FITC were purchased from Santa Cruz Biotechnology (CA USA). Phenylmethanesulfonyl fluoride (PMSF), sodium orthovanadate, sodium fluoride, and a protease inhibitor mixture were purchased from Roche Molecular Biochemicals (Indianapolis, IN, USA).

### Animal experiment

Care, use, and treatment of animals were done in agreement with the guidelines established by the Seoul National University Institutional Animal Care and Use Committee (SNU- 110415–2). Male BALB/c mice (4 week-old) were housed for 1 week before the experiments and maintained on a 12-h light/dark cycle, with food and water freely available. The mice were divided into the control (n = 6) and the treatment (n = 6) group. The treatment group mice were injected intraperitoneally with forskolin (20 μg/g), and the control mice received an equal volume of Dulbecco's Phosphate-Buffered Saline. After 6 h, the mice were exposed to whole body γ-ray irradiation (10 Gy).

### Expression constructs and transient transfection

H1299 cells were transfected with a EE-tagged constitutively active mutant (GαsQ227L, GαsQL) of long form stimulatory α subunit of G protein (Gαs) in a pcDNA3 vector (Invitrogen, Paisley, UK) using the calcium phosphate method [39]. A glutamine residue that is essential for the intrinsic GTPase activity is replaced with leucine in GαsQL [40]. A dominant negative mutant of PKA (dnPKA) was a gift from Dr. G. Stanley McKnight (University of Washington, WA, USA) [41]. Constitutively active mutant of I-kappa B kinase alpha S176E/S180E (IKKαSE) and beta S177E/181E (IKKβSE) were gifts from Dr. Dae-Myung Jue (The Catholic University of Korea) [42]. Small interfering RNAs (siRNAs) against ATM (cat. sc-29761) were purchased from Santa Cruz Biotechnology (CA, USA), and siRNA against PP2A B56δ (FlexiTube no. SI2653350) from Qiagen (Hilden, Germany). Control siRNA (5’-AATTCTCCGAACGTGTCACGT-3’) were purchased from Bioneer (Daejeon, Korea). siRNAs were transfected using Lipofectaimine (Invitrogen, Paisley, UK), and the cells were treated with other reagents at 48 h after transfection.

### Preparation of cytosolic and nuclear fractions

The cultured cells were harvested and then disrupted in lysis buffer A (0.33 M sucrose, 10 mM Hepes (pH 7.4), 1 mM MgCl_2_, 0.1% Triton X-100, protease inhibitor cocktail (PIC), and PMSF). The cell lysates were centrifuged for 5 min at 800 *g*, and the supernatants were collected to use as the cytosolic fractions. The resulting pellets were resuspended in lysis buffer B (0.45 M NaCl, 10 mM Hepes (pH 7.4), PIC, and PMSF) and centrifuged for 5 min at 20,000 *g*. The supernatants were collected to use as the nuclear fractions.

### Western blot analysis

Western blotting was performed as previously described [28]. Antibodies against Gαs, Ku70, ATM, COX-1, phosphorylated cAMP response element binding protein (p-CREB, Ser-133), PP2A B56δ, IκBα, p50 and p65 of NF-κB were obtained from Santa Cruz Biotechnology (CA, USA). Antibodies against Rad50, p-ATM (Ser-1981), γ-H2AX, Ku80, CREB, DNA-PKcs, poly (ADP-ribose) polymerase (PARP), cleaved caspase-3 (Asp-175), p-AKT (Ser-473), AKT, p-IκBα, and Myc-tag were obtained from Cell Signaling Technology (Beverly, MA, USA). An antibody against β-actin was purchased from Sigma (St. Louis, MO, USA), and an antibody against EE-tag was purchased from Covance (Princeton, NJ, USA). An antibody against phosphorylated B56δ (Ser-566) of protein phosphatase 2A (PP2A) was kindly provided by Dr. Paul Greengard (The Rockefeller University, New York) [23]. The proteins were visualized using the Enhanced Chemiluminescence (ECL) reagent (Thermo scientific, Waltham, MA) and detected using an LAS-3000 (R&D Systems, Inc. Minneapolis, MN, USA). The densities of the protein bands were quantified using the Multi Gauge v2.3 software (Fuji, Tokyo, Japan), and the relative band densities were expressed as ratios of the corresponding control densities.

### Immunofluorescence microscopy

H1299 cells were plated in 60 mm dishes and incubated until they became 60% confluent. The cells were transfected with vector or GαsQL plasmids, and after 24 h, they were irradiated with γ-rays (5 Gy) from a cesium (Cs) irradiator [15]. After 30 min, the cells were fixed with 4% paraformaldehyde for 20 min and permeated with 0.5% Triton X-100 for 10 min. After blocking with 2% BSA for 1 h, the cells were incubated overnight with an antibody against p-ATM (1:200) in 2% BSA, followed by incubation with goat anti-rabbit IgG-FITC (1:100) and DAPI (0.5 μl/ ml) for 1 h. The stained cells were observed with a confocal microscope (LSM 501 META, Carl Zeiss, Inc. USA).

### TUNEL assay

Extracted lung tissues from BALB/c mice were deparaffinized and hydrated. The tissues were stained using the ApopTag fluorescein *in situ* apoptosis detection kit (Chemicon International, Temecula, CA, USA), and apoptosis was observed using confocal laser scanning microscopy (TCS SP2, Leica, Wetzler, Germany).

### PP2A activity assay

Cells were prepared and lysed following the protocol of the PP2A activity assay kit (R&D Systems, Inc. Minneapolis, MN, USA). In brief, the cell lysates were incubated with Serine/Threonine Phosphatase substrate I for 30 min, and then, 10 μl of Malachite Green Reagent A was added and incubated for 10 min. Then, 10 μl of Malachite Green Reagent B was added and incubated for 20 min, and the absorbance at 620 nm was measured with the Benchmark Plus™ microplate reader (Bio-Rad, PA, USA).

### Flow cytometry

The cells were exposed to γ-rays (10 Gy) and incubated for 24 h. Then, the cells were washed twice with phosphate-buffered saline, harvested, and spun at 3,500 *g* for 5 min at 4°C. The cells were incubated in 1X Annexin V buffer containing Annexin V and PI for 15 min. Stained cells were quantified with a FacsCalibur flow cytometer (BD Biosciences, Franklin Lakes, NJ, USA) using 10,000 cells per measurement.

### Dual luciferase reporter assay

H1299 cells were transfected with plasmids containing luciferase reporter genes (NF-κB-pLuc and Renilla-pLuc) together with vector or GαsQL plasmids using the calcium phosphate method. Luciferase activities were measured using the Dual-Luciferase Reporter Assay System (Promega Corp., Madison, WI, USA) according to the manufacturer’s protocol. At least four independent experiments were performed in duplicate, and promoter activities were normalized using Renilla luciferase activity.

### Data analysis

At least three or more independent experiments were conducted for all the analyses, and the data were presented as the means ± standard errors (SE). The non-parametric Mann–Whitney U test was used to analyze the mean values, and a p value of less than 0.05 was considered statistically significant.

## Abbreviations

ATM: Ataxia–telangiectasia mutated; CREB: cAMP response element-binding protein; Gαs: Stimulatory α subunit of G protein; GαsQL: Constitutively active mutant long form of the α subunit of stimulatory heterotrimeric GTP binding protein; PKA: cAMP-dependent protein kinase; PP2A: Protein phosphatase 2A; PARP: Poly ADP (adenosine diphosphate)-ribose polymerase; qPCR: Quantitative polymerase chain reaction; siRNA: Small interfering RNA; TUNEL: Terminal uridine nucleotide end-labeling

## Competing interests

All authors declare that they have no competing interests.

## Authors’ contributions

EC and EK equally contributed in designing and performing experiments and wrote the manuscript together, and SK performed some of the experiments. YJ conceived experiments, participated in its design and coordination, edited manuscript, provided funding and resources required for conducting all experiments. All authors read and approved the final manuscript.

## Supplementary Material

Additional file 1: Figure S1 Effect of Gαs on the phosphorylation of ATM following g-ray irradiation in H1299 lung cancer cells. **Figure S2.** Effect of Gαs on the phosphorylation of ATM and downstream effectors following γ-ray irradiation in lung cancer cells. **Figure S3.** Effect of 6-benzoyl cAMP on γ-ray induced ATM phosphorylation in H1299 cells. **Figure S4.** Effect of γ-ray irradiation on the expression of GαsQL in H1299 cells. **Figure S5.** Effect of okadaic acid on the radiation-induced ATM phosphorylation. **Figure S6.** Effect of Gαs on PP2A B56δ phosphorylation. **Figure S7.** Effect of PKA inhibition on the phosphorylation of PP2A B56δ and ATM. **Figure S8.** Effects of Gαs on radiation-induced cleavage of caspase-3 and PARP in H1299 cells. **Figure S9.** Effect of Gαs on survival of γ-ray irradiated cells. **Figure S10.** Effects of prostaglandin E2 and isoproterenol on the cleavage of caspase 3 and PARP. **Figure S11.** Effect of H-89 on the inhibition of radiation-induced ATM phosphorylation by PGE2 and isoproterenol. **Figure S12.** Effects of prostaglandin E2 and isoproterenol on survival of γ-ray irradiated cells. Click here for file
