# Supplementary material for: cAMP signaling inhibits radiation-induced ATM phosphorylation leading to the augmentation of apoptosis in human lung cancer cells
Source: Mol Cancer. 2014 Feb 24;13:36. doi: 10.1186/1476-4598-13-36 (PMC4234305; doi:10.1186/1476-4598-13-36)
Supplement: Additional file 1: Figure S1 — Effect of Gαs on the phosphorylation of ATM following g-ray irradiation in H1299 lung cancer cells. Figure S2. Effect of Gαs on the phosphorylation of ATM and downstream effectors following γ-ray irradiation in lung cancer cells. Figure S3. Effect of 6-benzoyl cAMP on γ-ray induced ATM phosphorylation in H1299 cells. Figure S4. Effect of γ-ray irradiation on the expression of GαsQL in H1299 cells. Figure S5. Effect of okadaic acid on the radiation-induced ATM phosphorylation. Figure S6. Effect of Gαs on PP2A B56δ phosphorylation. Figure S7. Effect of PKA inhibition on the phosphorylation of PP2A B56δ and ATM. Figure S8. Effects of Gαs on radiation-induced cleavage of caspase-3 and PARP in H1299 cells. Figure S9. Effect of Gαs on survival of γ-ray irradiated cells. Figure S10. Effects of prostaglandin E2 and isoproterenol on the cleavage of caspase 3 and PARP. Figure S11. Effect of H-89 on the inhibition of radiation-induced ATM phosphorylation by PGE2 and isoproterenol. Figure S12. Effects of prostaglandin E2 and isoproterenol on survival of γ-ray irradiated cells. [file 1476-4598-13-36-S1.pptx]

## Slide 1
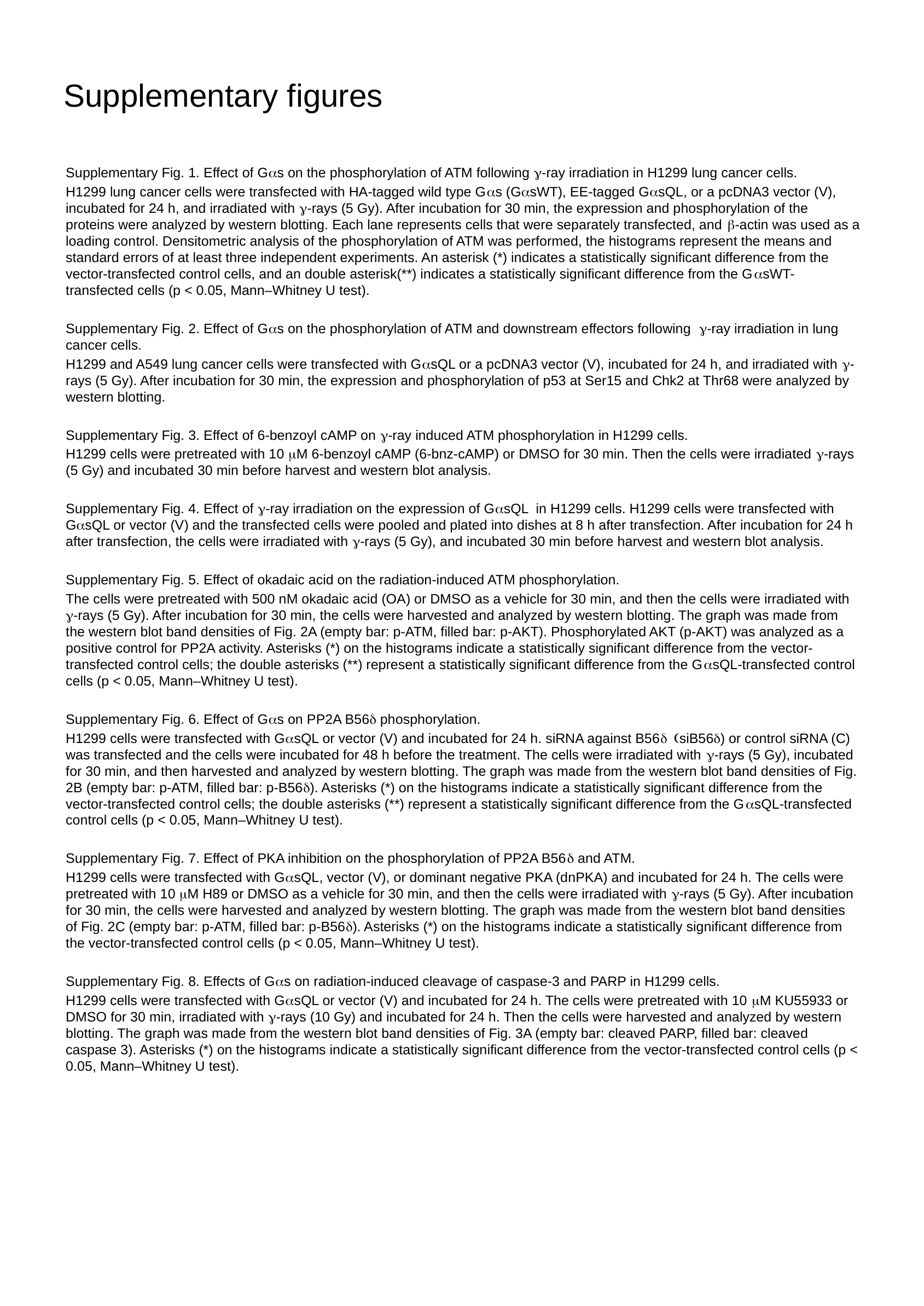

# Supplementary figures
Supplementary Fig. 1. Effect of Gas on the phosphorylation of ATM following g-ray irradiation in H1299 lung cancer cells.
H1299 lung cancer cells were transfected with HA-tagged wild type Gas (GasWT), EE-tagged GasQL, or a pcDNA3 vector (V), incubated for 24 h, and irradiated with g-rays (5 Gy). After incubation for 30 min, the expression and phosphorylation of the proteins were analyzed by western blotting. Each lane represents cells that were separately transfected, and b-actin was used as a loading control. Densitometric analysis of the phosphorylation of ATM was performed, the histograms represent the means and standard errors of at least three independent experiments. An asterisk (*) indicates a statistically significant difference from the vector-transfected control cells, and an double asterisk(**) indicates a statistically significant difference from the GasWT-transfected cells (p < 0.05, Mann–Whitney U test).
Supplementary Fig. 2. Effect of Gαs on the phosphorylation of ATM and downstream effectors following γ-ray irradiation in lung cancer cells.
H1299 and A549 lung cancer cells were transfected with GasQL or a pcDNA3 vector (V), incubated for 24 h, and irradiated with g-rays (5 Gy). After incubation for 30 min, the expression and phosphorylation of p53 at Ser15 and Chk2 at Thr68 were analyzed by western blotting.
Supplementary Fig. 3. Effect of 6-benzoyl cAMP on g-ray induced ATM phosphorylation in H1299 cells.
H1299 cells were pretreated with 10 mM 6-benzoyl cAMP (6-bnz-cAMP) or DMSO for 30 min. Then the cells were irradiated g-rays (5 Gy) and incubated 30 min before harvest and western blot analysis.
Supplementary Fig. 4. Effect of g-ray irradiation on the expression of GasQL in H1299 cells. H1299 cells were transfected with GasQL or vector (V) and the transfected cells were pooled and plated into dishes at 8 h after transfection. After incubation for 24 h after transfection, the cells were irradiated with g-rays (5 Gy), and incubated 30 min before harvest and western blot analysis.
Supplementary Fig. 5. Effect of okadaic acid on the radiation-induced ATM phosphorylation.
The cells were pretreated with 500 nM okadaic acid (OA) or DMSO as a vehicle for 30 min, and then the cells were irradiated with γ-rays (5 Gy). After incubation for 30 min, the cells were harvested and analyzed by western blotting. The graph was made from the western blot band densities of Fig. 2A (empty bar: p-ATM, filled bar: p-AKT). Phosphorylated AKT (p-AKT) was analyzed as a positive control for PP2A activity. Asterisks (*) on the histograms indicate a statistically significant difference from the vector-transfected control cells; the double asterisks (**) represent a statistically significant difference from the GαsQL-transfected control cells (p < 0.05, Mann–Whitney U test).
Supplementary Fig. 6. Effect of Gαs on PP2A B56 phosphorylation.
H1299 cells were transfected with GαsQL or vector (V) and incubated for 24 h. siRNA against B56 (siB56δ) or control siRNA (C) was transfected and the cells were incubated for 48 h before the treatment. The cells were irradiated with γ-rays (5 Gy), incubated for 30 min, and then harvested and analyzed by western blotting. The graph was made from the western blot band densities of Fig. 2B (empty bar: p-ATM, filled bar: p-B56d). Asterisks (*) on the histograms indicate a statistically significant difference from the vector-transfected control cells; the double asterisks (**) represent a statistically significant difference from the GαsQL-transfected control cells (p < 0.05, Mann–Whitney U test).
Supplementary Fig. 7. Effect of PKA inhibition on the phosphorylation of PP2A B56 and ATM.
H1299 cells were transfected with GαsQL, vector (V), or dominant negative PKA (dnPKA) and incubated for 24 h. The cells were pretreated with 10 μM H89 or DMSO as a vehicle for 30 min, and then the cells were irradiated with γ-rays (5 Gy). After incubation for 30 min, the cells were harvested and analyzed by western blotting. The graph was made from the western blot band densities of Fig. 2C (empty bar: p-ATM, filled bar: p-B56d). Asterisks (*) on the histograms indicate a statistically significant difference from the vector-transfected control cells (p < 0.05, Mann–Whitney U test).
Supplementary Fig. 8. Effects of Gαs on radiation-induced cleavage of caspase-3 and PARP in H1299 cells.
H1299 cells were transfected with GαsQL or vector (V) and incubated for 24 h. The cells were pretreated with 10 μM KU55933 or DMSO for 30 min, irradiated with γ-rays (10 Gy) and incubated for 24 h. Then the cells were harvested and analyzed by western blotting. The graph was made from the western blot band densities of Fig. 3A (empty bar: cleaved PARP, filled bar: cleaved caspase 3). Asterisks (*) on the histograms indicate a statistically significant difference from the vector-transfected control cells (p < 0.05, Mann–Whitney U test).

## Slide 2
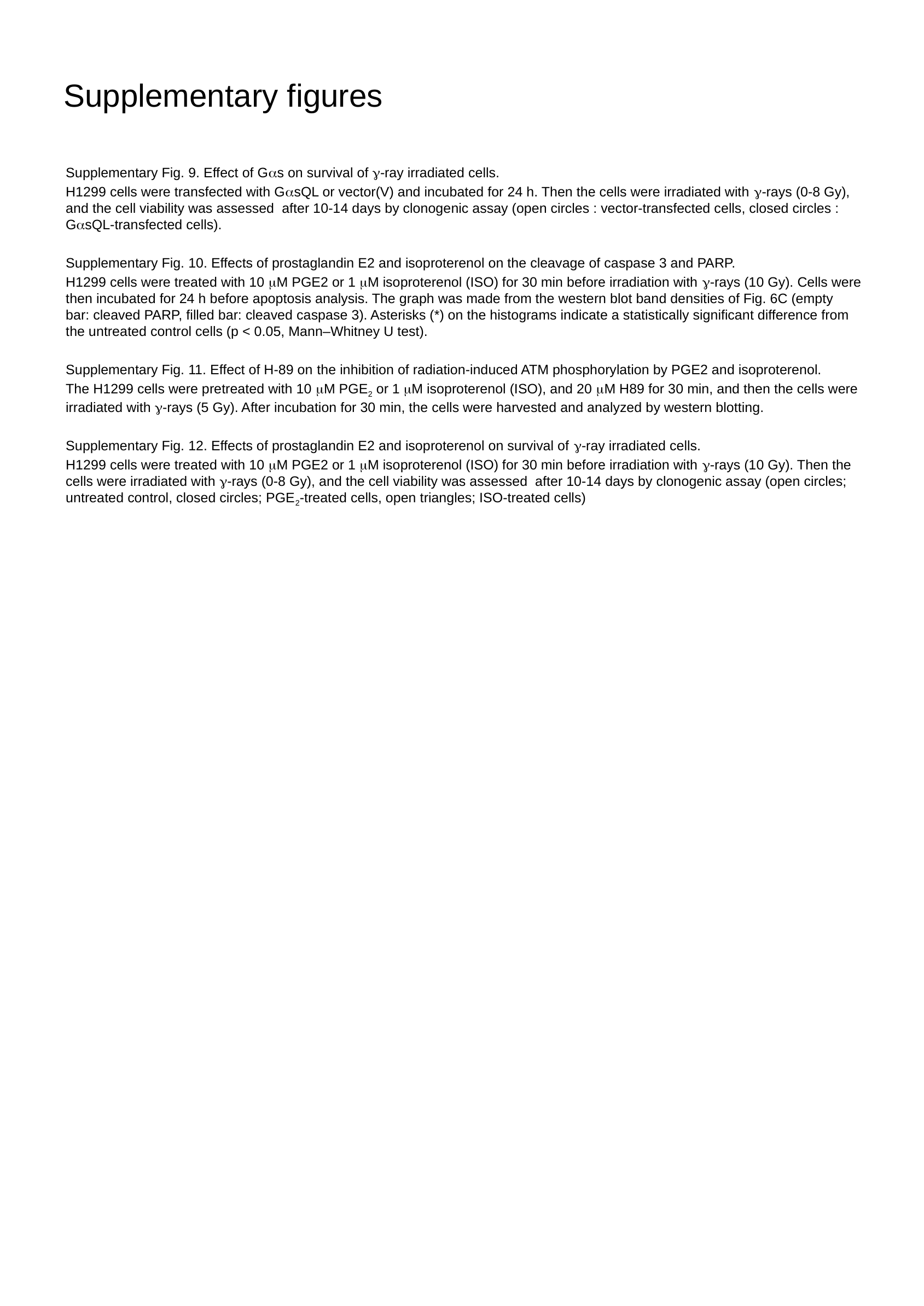

# Supplementary figures
Supplementary Fig. 9. Effect of Gas on survival of g-ray irradiated cells.
H1299 cells were transfected with GasQL or vector(V) and incubated for 24 h. Then the cells were irradiated with g-rays (0-8 Gy), and the cell viability was assessed after 10-14 days by clonogenic assay (open circles : vector-transfected cells, closed circles : GasQL-transfected cells).
Supplementary Fig. 10. Effects of prostaglandin E2 and isoproterenol on the cleavage of caspase 3 and PARP.
H1299 cells were treated with 10 μM PGE2 or 1 μM isoproterenol (ISO) for 30 min before irradiation with γ-rays (10 Gy). Cells were then incubated for 24 h before apoptosis analysis. The graph was made from the western blot band densities of Fig. 6C (empty bar: cleaved PARP, filled bar: cleaved caspase 3). Asterisks (*) on the histograms indicate a statistically significant difference from the untreated control cells (p < 0.05, Mann–Whitney U test).
Supplementary Fig. 11. Effect of H-89 on the inhibition of radiation-induced ATM phosphorylation by PGE2 and isoproterenol.
The H1299 cells were pretreated with 10 mM PGE2 or 1 mM isoproterenol (ISO), and 20 mM H89 for 30 min, and then the cells were irradiated with g-rays (5 Gy). After incubation for 30 min, the cells were harvested and analyzed by western blotting.
Supplementary Fig. 12. Effects of prostaglandin E2 and isoproterenol on survival of g-ray irradiated cells.
H1299 cells were treated with 10 μM PGE2 or 1 μM isoproterenol (ISO) for 30 min before irradiation with γ-rays (10 Gy). Then the cells were irradiated with g-rays (0-8 Gy), and the cell viability was assessed after 10-14 days by clonogenic assay (open circles; untreated control, closed circles; PGE2-treated cells, open triangles; ISO-treated cells)

## Slide 3
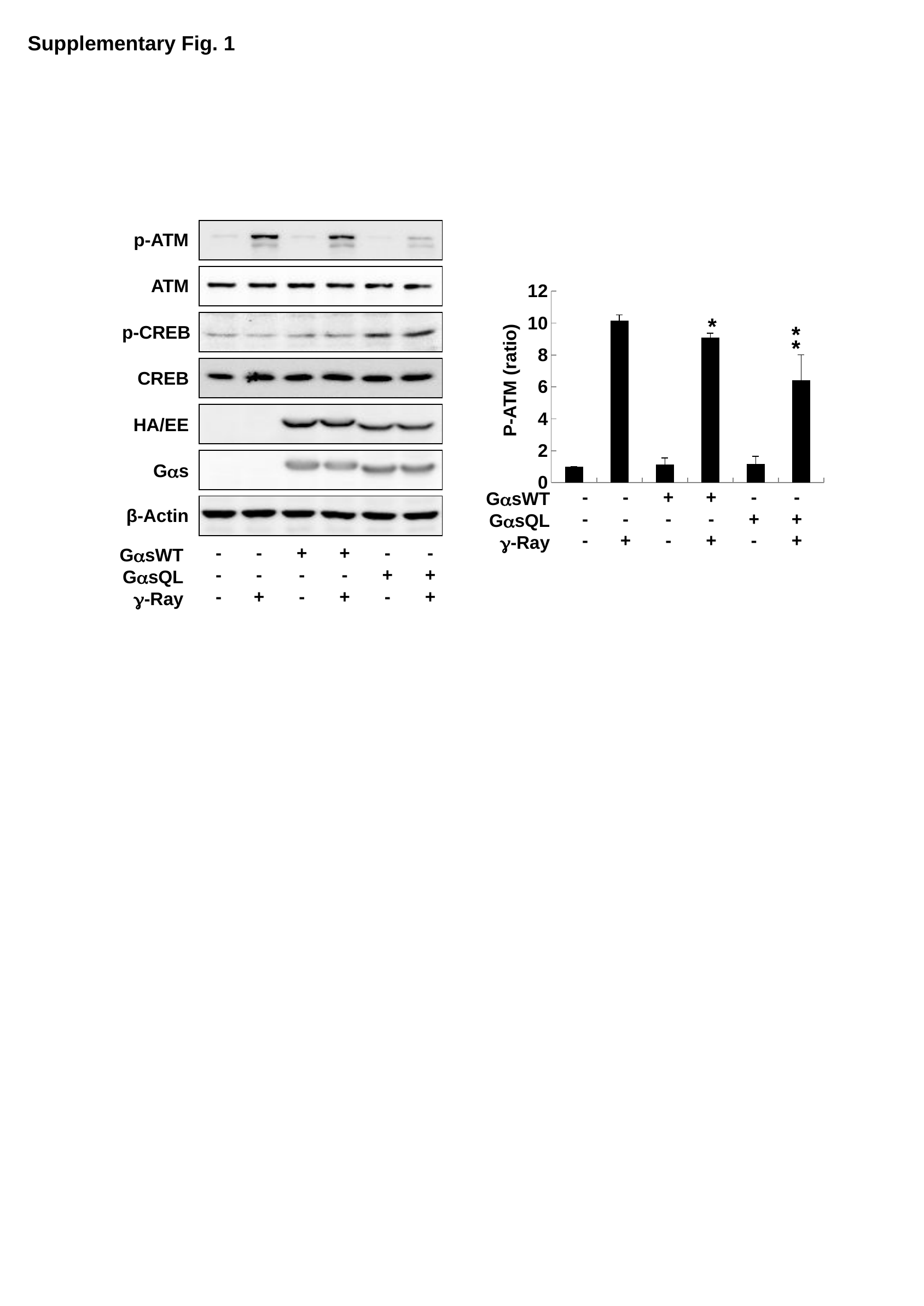

Supplementary Fig. 1
p-ATM
ATM
p-CREB
CREB
HA/EE
Gas
β-Actin
-
-
-
-
-
+
+
-
-
+
-
+
-
+
-
-
+
+
GasWT
GasQL
g-Ray
### Chart
| Category | |
|---|---|*
*
*
P-ATM (ratio)
-
-
-
-
-
+
+
-
-
+
-
+
-
+
-
-
+
+
GasWT
GasQL
g-Ray

## Slide 4
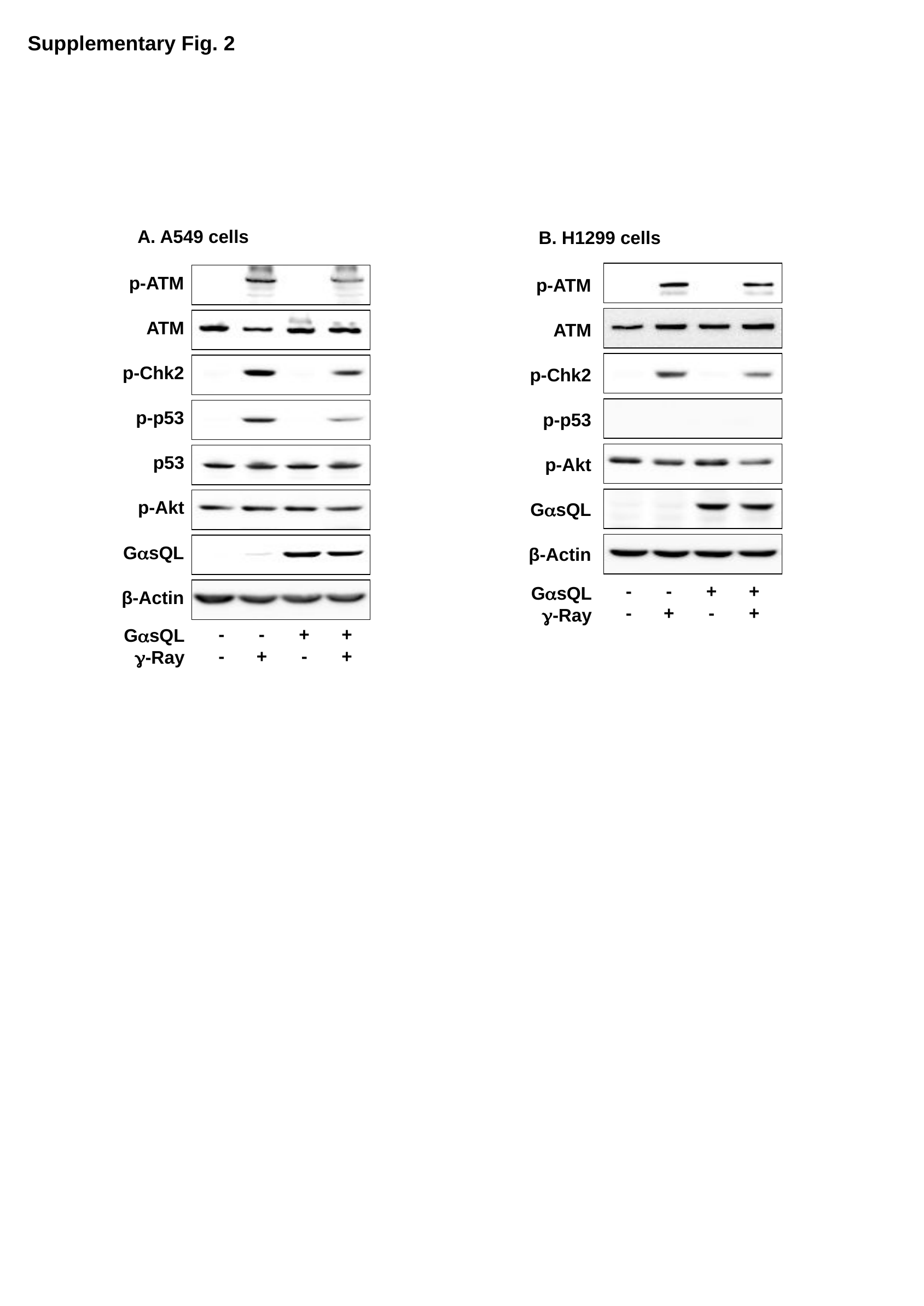

Supplementary Fig. 2
A. A549 cells
p-ATM
ATM
p-Chk2
p-p53
p53
p-Akt
GasQL
β-Actin
-
-
-
+
+
-
+
+
GasQL
g-Ray
B. H1299 cells
p-ATM
ATM
p-Chk2
p-p53
p-Akt
GasQL
β-Actin
-
-
-
+
+
-
+
+
GasQL
g-Ray

## Slide 5
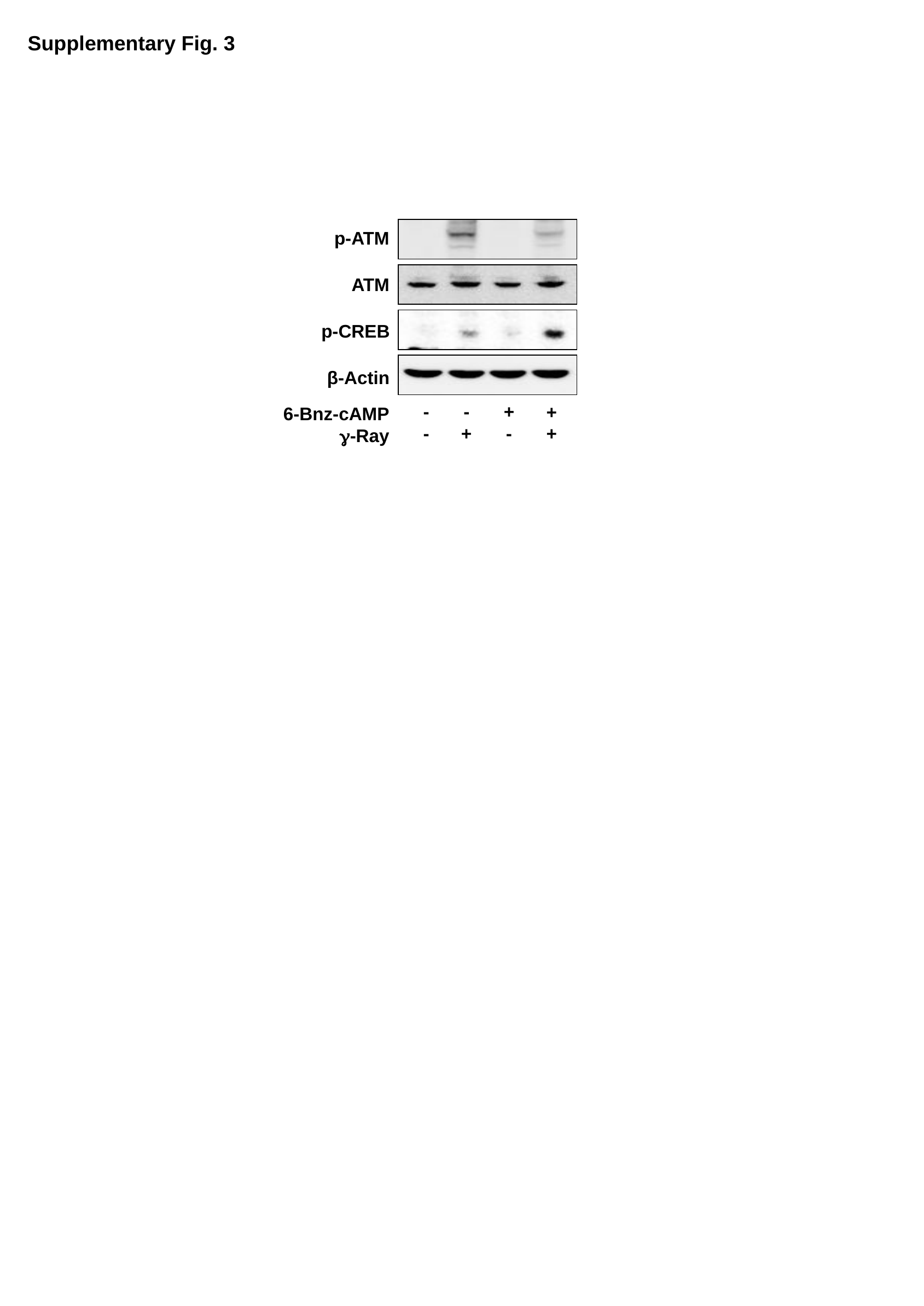

Supplementary Fig. 3
p-ATM
ATM
p-CREB
β-Actin
-
-
-
+
+
-
+
+
6-Bnz-cAMP
g-Ray

## Slide 6
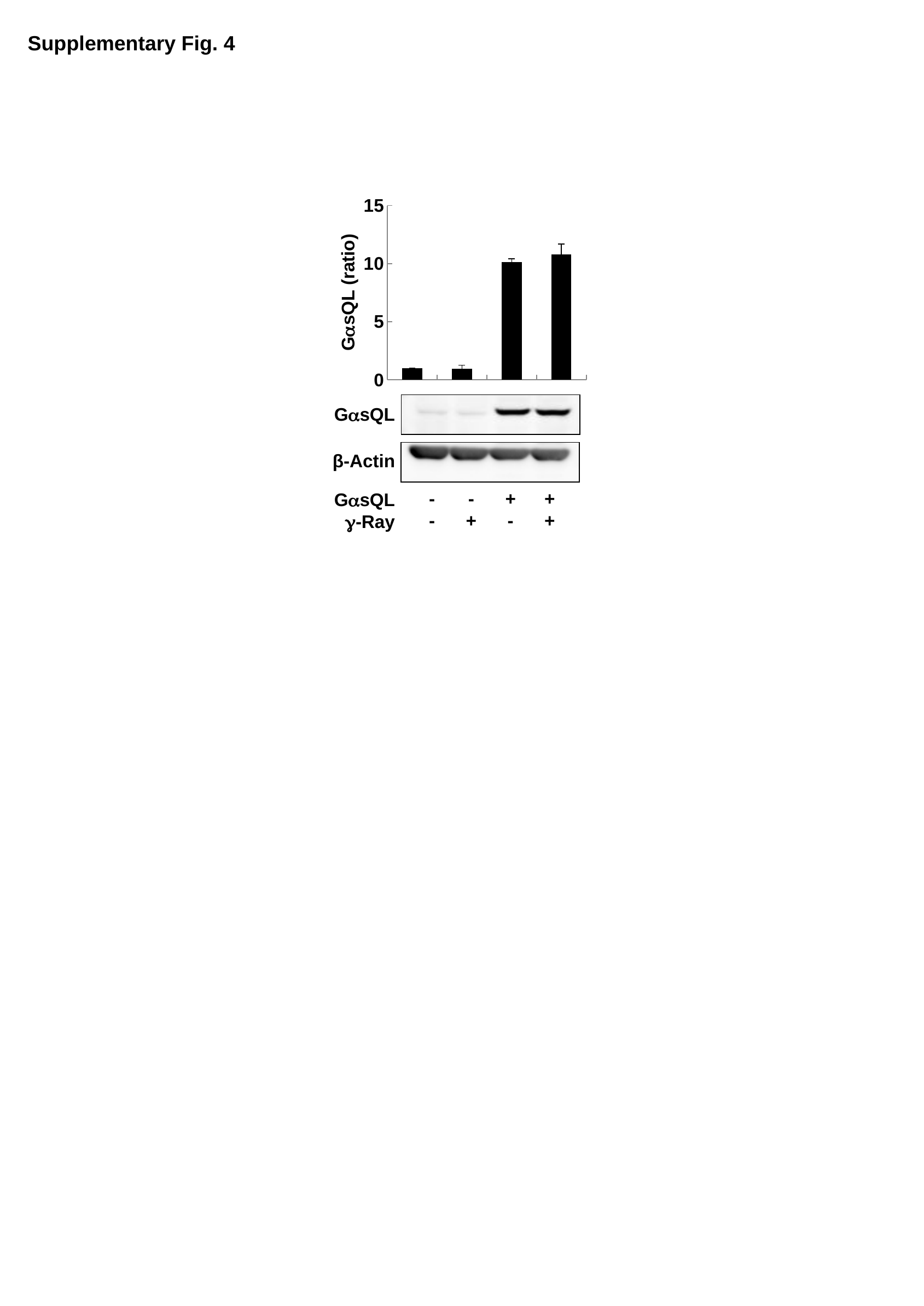

Supplementary Fig. 4
### Chart
| Category | |
|---|---|
| V | 1.0 |
| V+R | 0.9666666666666667 |
| G | 10.143333333333333 |
| G+R | 10.816666666666668 |GasQL (ratio)
GasQL
β-Actin
+
-
+
+
-
-
-
+
GasQL
g-Ray

## Slide 7
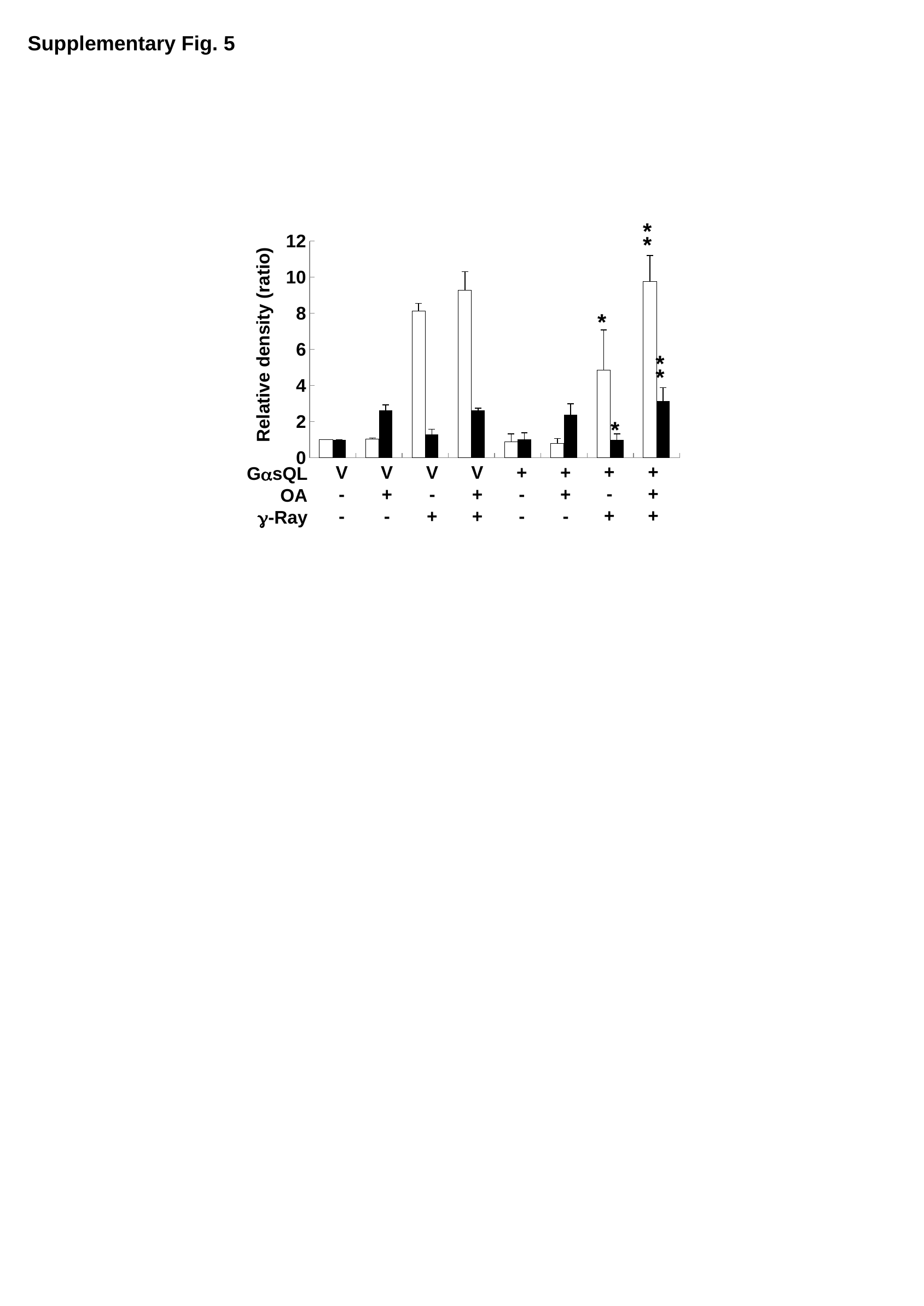

Supplementary Fig. 5
### Chart
| Category | | |
|---|---|---|*
Relative density (ratio)
*
+
-
+
+
+
+
+
-
-
+
+
-
V
-
-
V
+
-
V
-
+
V
+
+
GasQL
OA
g-Ray
*
*
*
*

## Slide 8
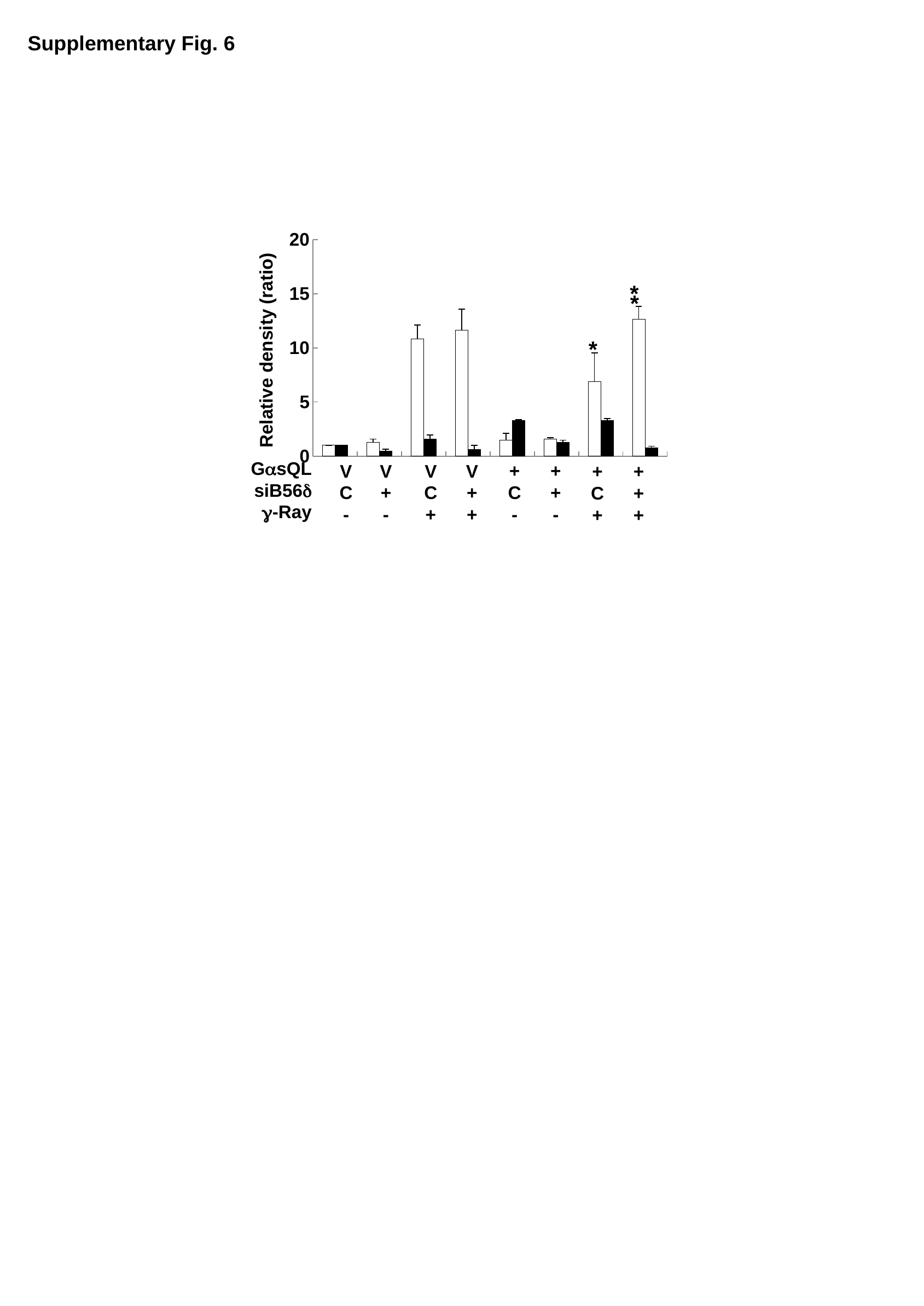

Supplementary Fig. 6
### Chart
| Category | | |
|---|---|---|*
*
*
Relative density (ratio)
GasQL
siB56d
 g-Ray
+
C
-
+
+
-
V
C
-
V
+
-
V
C
+
V
+
+
+
C
+
+
+
+

## Slide 9
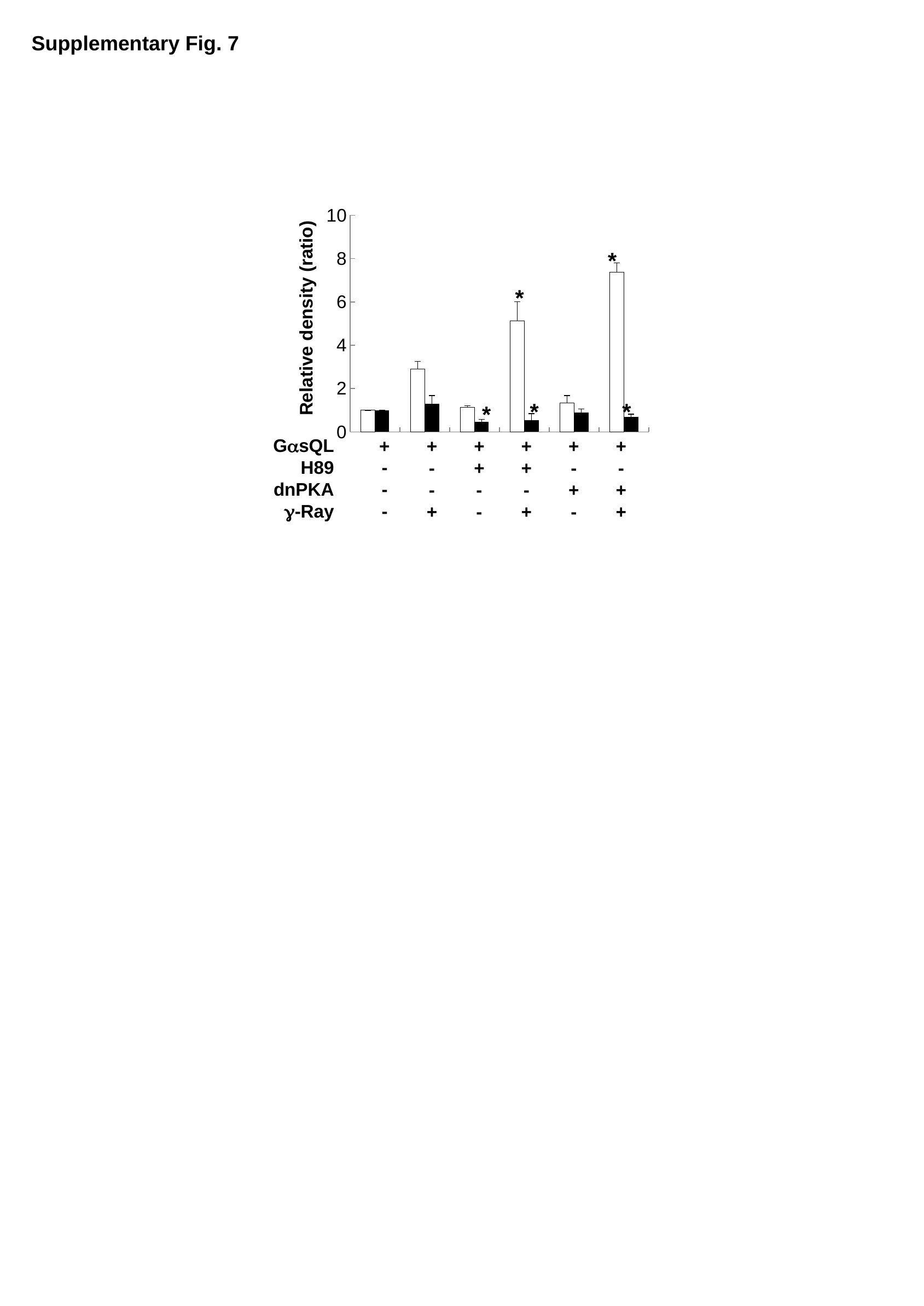

Supplementary Fig. 7
### Chart
| Category | | |
|---|---|---|*
*
Relative density (ratio)
*
*
*
GasQL
H89
dnPKA
g-Ray
+
-
-
-
+
-
-
+
+
+
-
-
+
+
-
+
+
-
+
-
+
-
+
+

## Slide 10
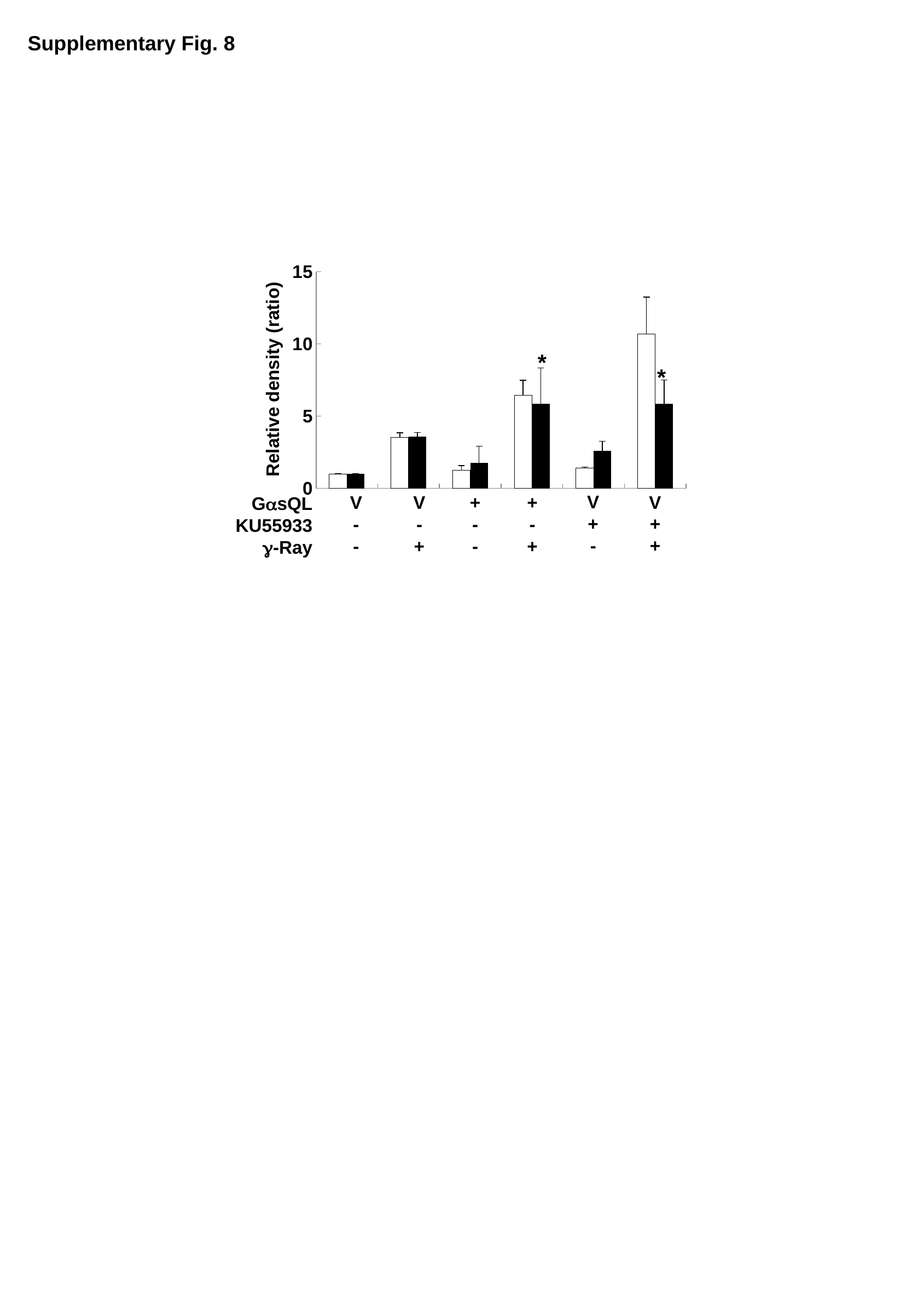

Supplementary Fig. 8
### Chart
| Category | | |
|---|---|---|*
*
Relative density (ratio)
V
+
-
V
+
+
V
-
-
V
-
+
+
-
-
+
-
+
GasQL
KU55933
g-Ray

## Slide 11
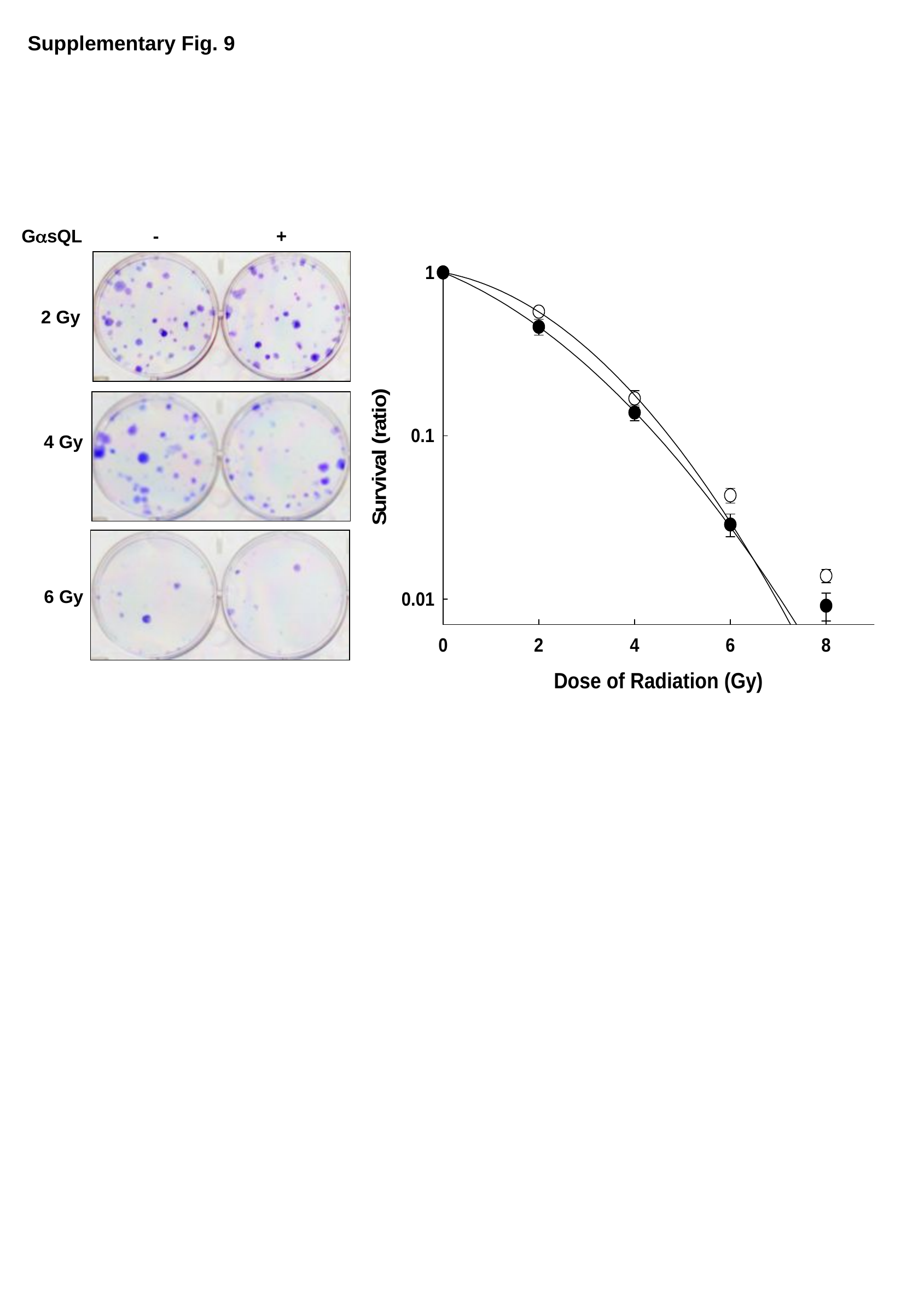

Supplementary Fig. 9
GasQL
-
+
2 Gy
4 Gy
6 Gy

## Slide 12
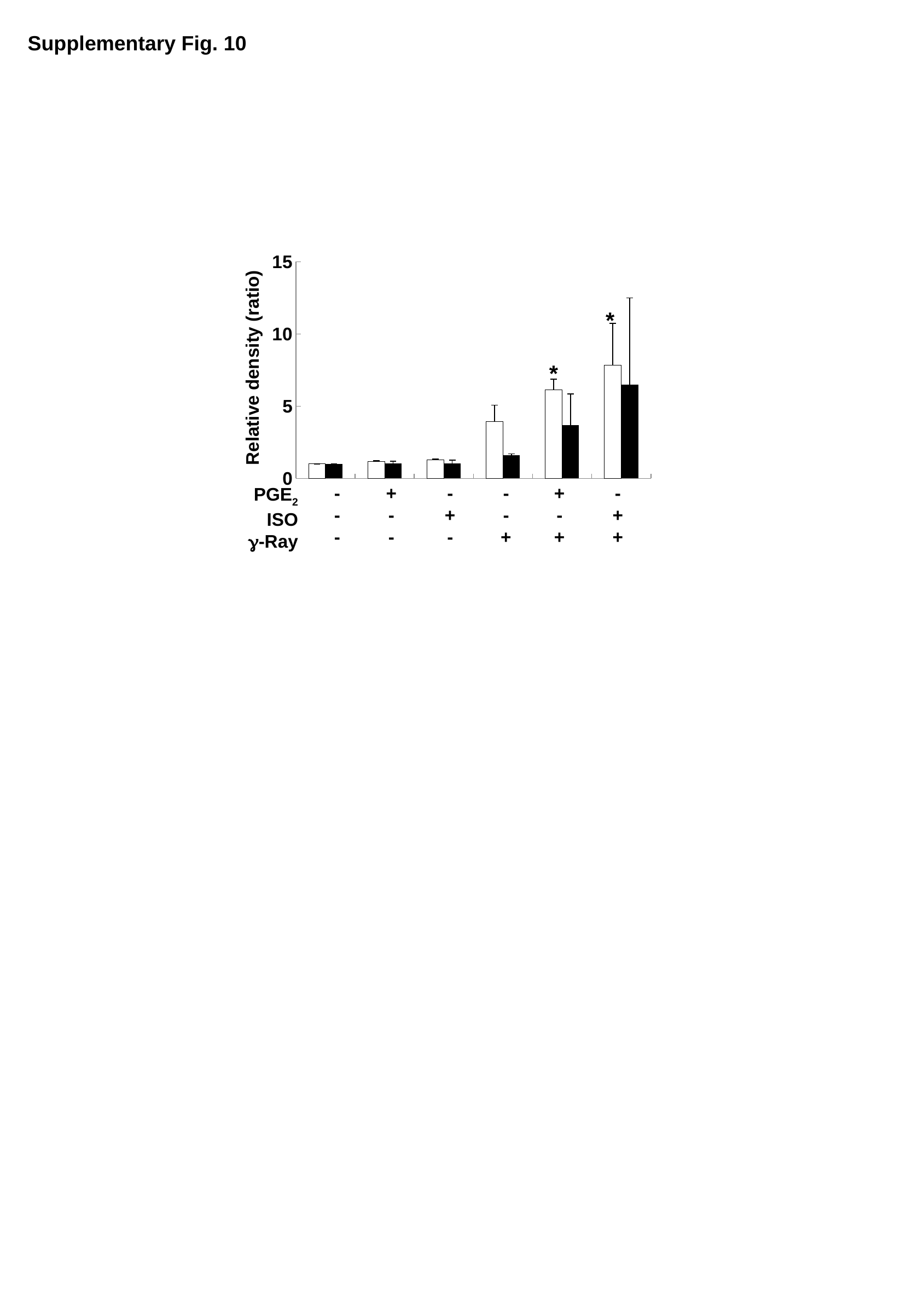

Supplementary Fig. 10
### Chart
| Category | | |
|---|---|---|*
Relative density (ratio)
*
+
-
+
-
+
+
-
-
-
+
-
-
-
+
-
-
-
+
PGE2
ISO
g-Ray

## Slide 13
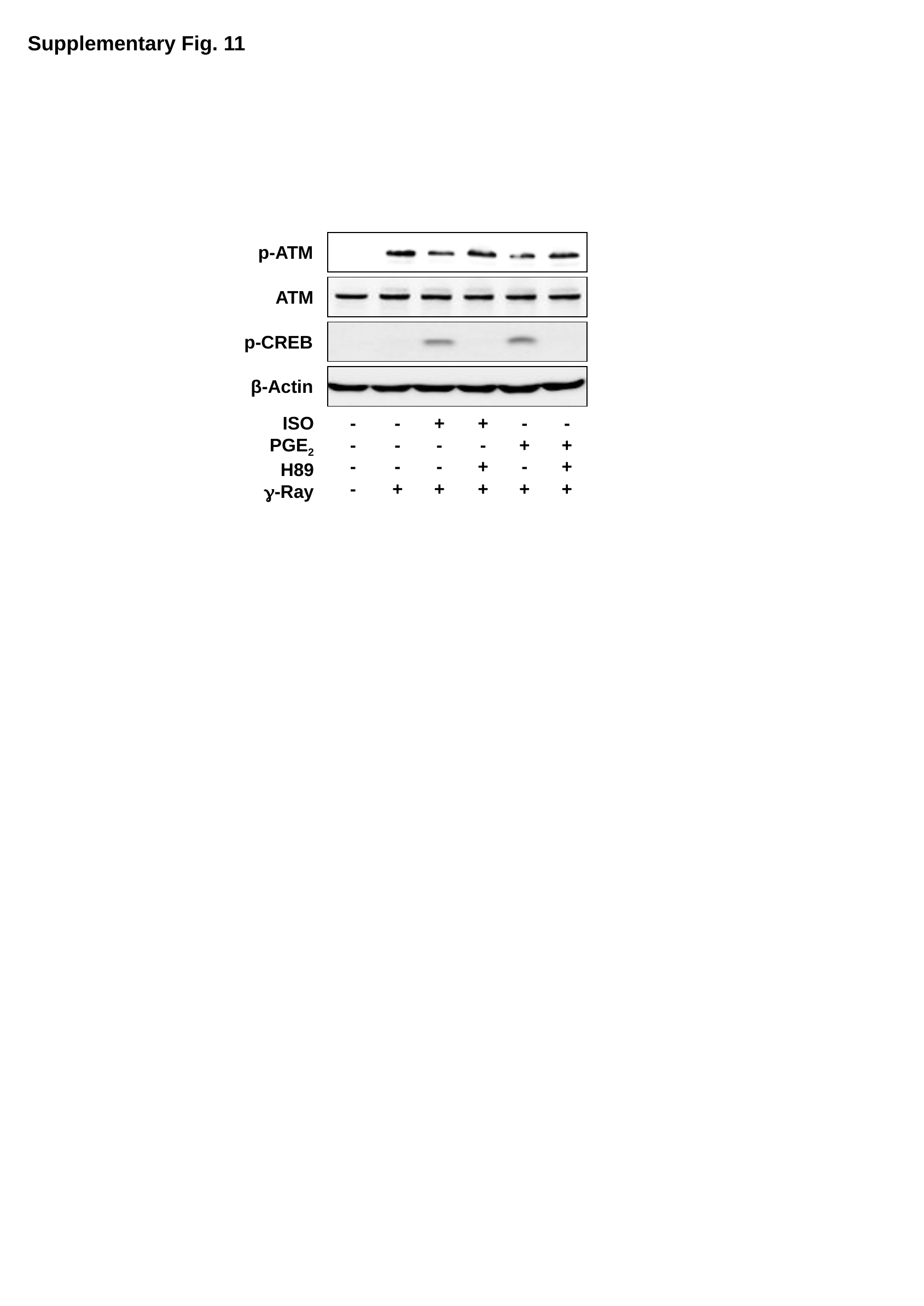

Supplementary Fig. 11
p-ATM
ATM
p-CREB
β-Actin
ISO
PGE2
H89
g-Ray
-
-
-
-
-
-
-
+
+
-
-
+
+
-
+
+
-
+
-
+
-
+
+
+

## Slide 14
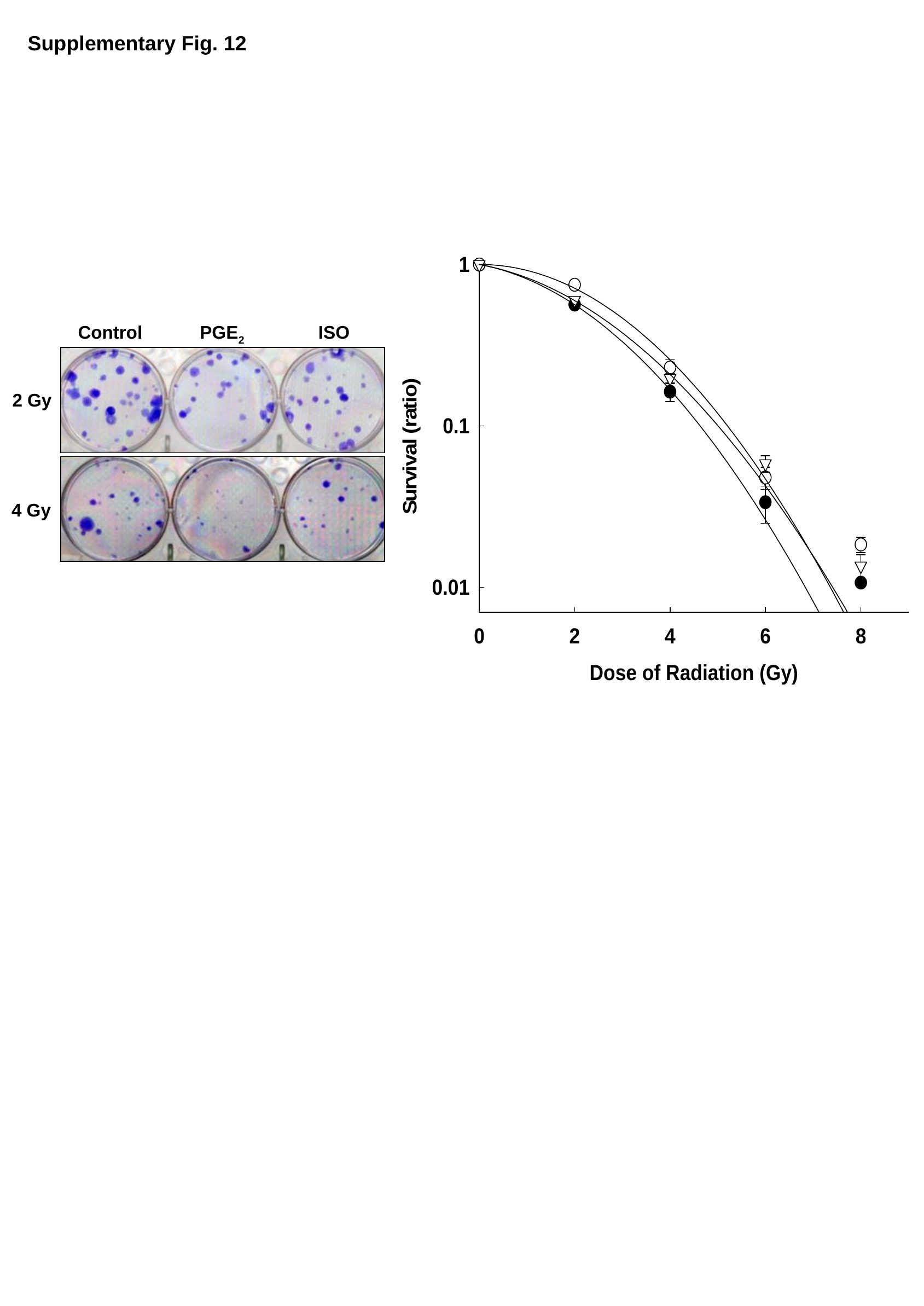

Supplementary Fig. 12
Control
PGE2
ISO
2 Gy
4 Gy
